# The potential linkage between sediment oxygen demand and microbes and its contribution to the dissolved oxygen depletion in the Gan River

**DOI:** 10.3389/fmicb.2024.1413447

**Published:** 2024-07-31

**Authors:** Shoutao Cheng, Fansheng Meng, Yeyao Wang, Jiasheng Zhang, Lingsong Zhang

**Affiliations:** ^1^Country School of Water Resources and Environment, China University of Geosciences (Beijing), Beijing, China; ^2^Research Center of Environmental Pollution Control Technology, Chinese Research Academy of Environmental Sciences, Beijing, China; ^3^China National Environmental Monitoring Center, Beijing, China

**Keywords:** dissolved oxygen depletion, sediment, sediment oxygen demand, putative functions of sediment microbes, Gan River

## Abstract

The role of sediment oxygen demand (SOD) in causing dissolved oxygen (DO) depletion is widely acknowledged, with previous studies mainly focusing on chemical and biological SOD separately. However, the relationship between the putative functions of sediment microbes and SOD, and their impact on DO depletion in overlying water, remains unclear. In this study, DO depletion was observed in the downstream of the Gan River during the summer. Sediments were sampled from three downstream sites (YZ, Down1, and Down2) and one upstream site (CK) as a control. Aquatic physicochemical parameters and SOD levels were measured, and microbial functions were inferred from taxonomic genes through analyses of the 16S rRNA gene. The results showed that DO depletion sites exhibited a higher SOD rate compared to CK. The microbial community structure was influenced by the spatial variation of Proteobacteria, Chloroflexi, and Bacteroidota, with total organic carbon (TOC) content acting as a significant environmental driver. A negative correlation was observed between microbial diversity and DO concentration (*p* < 0.05). Aerobic microbes were more abundant in DO depletion sites, particularly Proteobacteria. Microbes involved in various biogeochemical cycles, such as carbon (methane oxidation, methanotrophs, and methylotrophs), nitrogen (nitrification and denitrification), sulfur (sulfide and sulfur compound oxidation), and manganese cycles (manganese oxidation), exhibited higher abundance in DO depletion sites, except for the iron cycle (iron oxidation). These processes were negatively correlated with DO concentration and positively with SOD (*p* < 0.05). Overall, the results highlight that aerobic bacteria’s metabolic processes consume oxygen, increasing the SOD rate and contributing to DO depletion in the overlying water. Additionally, the study underscores the importance of targeting the removal of *in situ* microbial molecular mechanisms associated with toxic H_2_S and CH_4_ to support reoxygenation efforts in rehabilitating DO depletion sites in the Gan River, aiding in identifying factors controlling DO consumption and offering practical value for the river’s restoration and management.

## 1 Introduction

Dissolved oxygen (DO) refers to the concentration of oxygen gas in aquatic environments, garnering attention due to its vital role in maintaining well-oxygenated habitats for aquatic organisms’ survival, as well as regulating metabolic activities and subsequent trophodynamics (Mattson et al., 2008; Li et al., 2020). The presence of hypoxia (DO <2 mg/L) in rivers significantly impacts aquatic organisms (Shen et al., 2023), not only disrupts ecological balance by altering fish behavior and distribution (Moriarty et al., 2020), but also alters both structure and function of benthic communities (Levin et al., 2009). Over recent decades, numerous studies have primarily focused on hypoxia formation in rivers, encompassing two key aspects: (i) Production: DO in aquatic water primarily derives from atmospheric sources and photosynthesis by aquatic plants; (ii) Consumption: DO is mainly influenced by respiration, biological and chemical oxygen demand, decomposition of organic matter, and sediment oxygen consumption (Banerjee et al., 2019). The balance of DO levels is influenced by various physical and chemical factors, including temperature, atmospheric pressure, pH, and hydrodynamic water volume. These factors can directly or indirectly interact or combine to affect DO concentration (Banerjee et al., 2019; Li et al., 2020; Zhang et al., 2022).

The sediment oxygen demand (SOD) is defined as the removal of DO from the overlying water by bottom sediments. It is widely acknowledged that SOD plays a significant role in DO depletion and exerts a substantial influence on the DO cycle in rivers (Rong and Shan, 2016; Lee et al., 2018). The SOD rate serves as a crucial indicator for assuming and estimating DO depletion, which can contribute to more than 50% of the total oxygen demand in the overlying water of rivers (Matlock et al., 2003; Rong and Shan, 2016). SOD comprises chemical sediment oxygen demand (CSOD) and biological sediment oxygen demand (BSOD), based on the specific type of oxygen consumption occurring within the bottom sediment (Rong and Shan, 2016). CSOD formation occurs through the oxidation processes of iron (Fe^2+^), manganese (Mn^2+^), and sulfur (S^2−^) in the sediment (Matzinger et al., 2010). The term BSOD typically refers to the oxygen demand of all organisms residing in the sediment, encompassing both benthic organism respiration and microbial decomposition of organic matter (Higashino et al., 2004). In a prior study, researchers focused on individually detecting total SOD, biological SOD, and chemical SOD to ascertain the relative contributions of biological SOD and chemical SOD to the total SOD (Rong and Shan, 2016).

Sediment plays a crucial role in the aquatic ecosystem, serving as both a sink and source of contaminants and various nutrients (Chiaia-Hernández et al., 2022). Microbes inhabiting river sediment contribute to ecosystem services and influence the balance and functioning of the ecosystem (Zhang et al., 2019). Microbial composition and abundance are influenced by a multitude of complex environmental factors, including nutrients and organic matter (Bryant et al., 2012). Moreover, the spatial heterogeneity of physicochemical properties along the river may also affect the structure and diversity of microbial communities (Ge et al., 2021). However, further investigation is required to explore the potential mechanisms connecting DO and biogeochemical responses in sediment (Liu et al., 2021).

The role of microorganisms in river ecosystems is crucial for biogeochemical processes, including the cycling of carbon, nitrogen, sulfur, iron, and manganese (Yang et al., 2022). These metabolic processes lead to oxygen depletion (Broman et al., 2017) and promote the SOD rate, resulting in anoxic conditions in both sediments and the overlying bottom water. The available electron acceptors for microbial communities in sediments are ranked in descending order of energy gain: O_2_, NO_3_^−^, Mn^3+^, and Mn^4+^ oxides, Fe^3+^ oxides, and SO_4_^2−^. This process affects the oxygen reduction potential of sediments (Armstrong-Altrin et al., 2022). The aerobic microbes thrive when DO is abundant in the water–sediment interface, where they degrade nutrients, while anaerobic communities dominate when DO is depleted by aerobic organisms (Broman et al., 2017). Recently, high-throughput sequencing has provided a powerful tool to characterize river sediment-associated microbial community structure, diversity, and relative abundance by using the V3–V4 region of the bacterial 16S rRNA gene for sequencing (Yang et al., 2022). The 16S rDNA amplicon sequencing approach offers an opportunity to investigate the spatial distribution of microbial compositions in river sediments while exploring the effects of sediment physiochemical properties on this variation (Ge et al., 2021). Additionally, this method can be employed to investigate microbial communities in lake sediments and elucidate their putative biogeochemical functions (Kumar et al., 2019). However, there still exists a gap regarding the relationship between SOD and the putative metabolic and ecological functions of sediment microbes in rivers and their contribution to DO depletion.

In this study, downstream sites (YZ, Down1, and Down2) of the Gan River were selected because the occurrence of DO depletion phenomenon was observed in the summer, while an upstream site with normal DO levels was selected as a control site (CK). Sediment microbial communities’ structure and diversity were analyzed by high-throughput sequencing (HTS), and their putative functional diversity and main functional pathways were predicted using BugBase, PICRUSt2, and FAPROTAX platforms. In this study, we attempt to answer the following questions:

1. Does sediment microbial community structure along the Gan River change under different physicochemical characteristics of overlying water and sediment?
2. How do physicochemical characteristics affect the composition of microbial communities?
3. Additionally, is there any clear relationship between the functional diversity of microbial communities in terms of high oxygen demand metabolic pathways and SOD rates?

## 2 Materials and methods

### 2.1 Sampling sites

**Figure 1:**
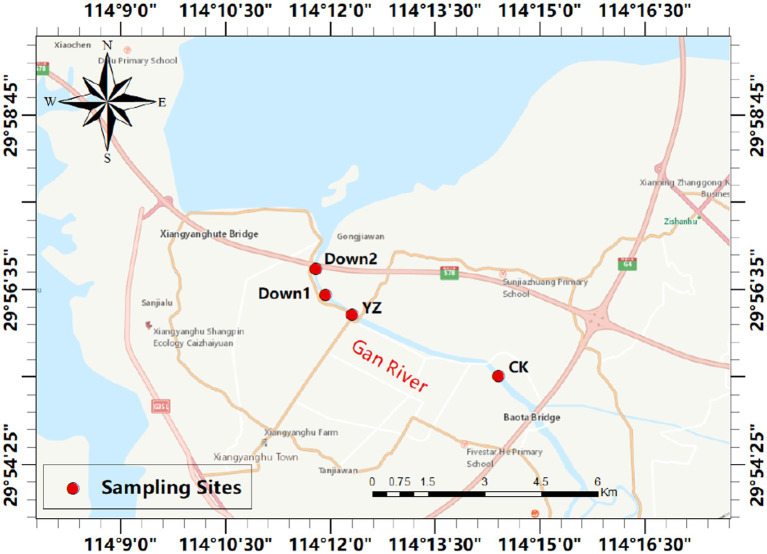
The map of four sampling sites selected in Gan River, China. The red circles indicate the sampling sites (see Supplementary Table S1 for details).

The Gan River, located in southern China, serves as a tributary of the Yangtze River. From April to October 2022, the dissolved oxygen concentration in the Yaozui Bridge (YZ) section was lower than 5 mg/L. In August 2022, we conducted sampling at four sites along the river, prompted by a decrease in dissolved oxygen (DO) observed at the Yaozui Bridge (YZ) section (Figure 1). The CK, serving as a control site, is situated 5 km upstream of YZ, while Down1 and Down2 are positioned 1 km and 2 km downstream of YZ, respectively. Further details regarding the sampling site locations are provided in Supplementary Table S1.

### 2.2 Water and sediment sampling

The water at the sediment–water interface was collected using a 5 L plexiglass water sampler (PSC-1A, Changzhou Pun Sen Electronic Instrument Factory, Changzhou, China) and subsampled into three sterile plastic bottles (500 mL each). One of the bottles had concentrated sulfuric acid added to it. Immediately after sampling, all bottles were sealed and transported back to the laboratory for storage at 4°C. The water sampler was sterilized with sodium hypochlorite (35%) for half an hour between sampling sites, followed by rinsing with river water three times at the next site. Sediment samples were collected using a dredge sampler, with a sampling depth ranging from 0 to 15 cm within the surface sediment. From each of the four sampling points, three replicate samples were collected, resulting in a total of 12 discrete sediment samples. Each sample was carefully sealed in sterile 2 L plastic boxes. These samples were sealed into sterile plastic boxes (2 L). Similar to the water sampler, the dredge sampler was sterilized with sodium hypochlorite (35%) for half an hour between sampling sites and then rinsed with river water three times at the next site. Upon returning to the laboratory, three replicate samples from each sampling site were homogenized and redistributed into three parts for different analyses. One part was transferred into a sterile 50-ml centrifuge tube for microbial community structure analysis; another part was reserved for detecting SOD; and the third sealed box was used for analyzing characteristic parameters (total nitrogen, ammonium nitrogen, total organic carbon, total phosphorus, and sulfide). All sediment samples were stored at −20°C before analysis.

### 2.3 Physicochemical characteristics of overlying water and sediment

The physicochemical characteristics of both overlying water and sediment were assessed within 1 week after sample collection using standard methods of China’s national environmental protection standards.

The following physicochemical characteristics of water samples were measured. Dissolved oxygen (DO, mg/L), temperature (°C), electrical conductivity (EC, μS/cm), total dissolved solids (TDS, mg/L), pH, and oxidation–reduction potential (ORP, mV) were measured using an HQ30d portable meter (HACH Company, Loveland, CO, USA) at a depth of 0.5 m beneath the surface of the river. Additionally, the water flow rate (WFR, m/s) was measured using an LS300-A portable meter (Runsun Instruments Inc., Chengdu, China). TOC was analyzed using a total organic carbon analyzer (TOC-VCPN, Shimadzu, Japan) (HJ/T 104-2003, China). Chemical oxygen demand (COD, mg/L) was measured using the dichromate method (HJ 828-2017, China). The permanganate index (COD_Mn_, mg/L) was measured (GB 118920-98, China). Five-day biochemical oxygen demand (BOD_5_, mg/L) was measured using the dilution and seeding method (HJ 505-2009, China). Total nitrogen (TN, mg/kg), ammonium nitrogen (NH_4_^+^-N, mg/kg), and total organic carbon (TOC, mg/kg) were measured using the alkaline potassium persulfate digestion UV spectrophotometric method (HJ 636-2012, China), Nessler’s reagent spectrophotometry method (HJ 535-2009, China), and ion chromatography method (HJ 84-2016), respectively. Total phosphorus (TP, mg/kg) was determined via the ammonium molybdate spectrophotometric method (GB 11893-1989, China).

The following physicochemical characteristics of sediment samples were measured. TOC (%) was determined using the potassium dichromate oxidation spectrophotometric method (HJ 615-2011, China). TN (mg/kg) was measured with the modified Kjeldahl method (HJ 717-2014, China). NH_4_^+^-N (mg/kg) was measured with potassium chloride solution-spectrophotometric methods (HJ 634–2012, China). TP (mg/kg) was measured using the alkali fusion Mo–Sb anti-spectrophotometric method (HJ 632-2011, China). Sulfide (mg/kg) was assessed using the methylene blue spectrophotometric method (HJ 833-2017, China). Depth measurements for both water and sediment were taken using a calibrated metal rod inserted into the river.

### 2.4 Sediment oxygen demand (SOD) measurement

The sediment oxygen demand (SOD) was promptly measured using the cylinder culture method upon transportation of the samples to the laboratory (Ling et al., 2009). At each of the four sampling sites, sediment samples underwent three incubation experiments. A total of 12 cylindrical Plexiglass incubation tubes (28 cm in length, 6 cm in diameter) were inserted approximately 10 cm into the sediment box, carefully extracted, and sealed with rubber stoppers at both ends. Subsequently, the tubes were meticulously filled with bottom water collected from the site (as closely as possible to the sediment) using a siphon. Additionally, four cylindrical Plexiglass incubation tubes were filled solely with the bottom water collected from the site, serving as controls. Electrodes were employed to record the DO concentration of overlying water every half an hour. These electrodes were inserted into the overlying water and connected to a DO meter through a hole in the upper rubber stopper. These tests were conducted in a laboratory environment maintained at a controlled temperature of 20°C using an air conditioner.

SOD (g m^−2^ d^−1^) was calculated based on a DO declining rate after incubation according to the following general Equation (1) (Lee et al., 2018):


(1) $$\mathrm{SOD}=\frac{dC}{dt}\times \frac{V}{A}\times \frac{24\;h}{1\mathrm{day}}\times \frac{1\;g}{1,000\;\mathrm{mg}}$$

where *dC*/*dt* (mg L^−1^ h^−1^) is the slope of the oxygen depletion curve, which was obtained using the best linear model; *V* (L) is the volume of overlying water in the cylinder; and *A* (m^2^) is the bottom area of a cross-section of sediment in the tube cylinder.

### 2.5 Microbial DNA extraction from sediment

The total genomic DNA was extracted from each frozen sediment sample (0.25 g) with a FastDNA Spin Kit (MP Biomedicals, Santa Ana, CA, USA) according to the manufacturer’s protocol. This is an extraction method frequently used for this purpose (Ge et al., 2021). The concentration and purity of the extracted DNA were determined on Qubit^®^ 4 Fluorometer (Invitrogen^®^) with the Qubit^®^ dsDNA HS Assay Kit (Invitrogen^®^). All the sediment DNA samples were extracted by the same person and stored at −20°C before amplification and sequencing analysis.

### 2.6 16S rRNA gene amplification and sequencing

The 16S rRNA gene was amplified in a 20 μL PCR reaction from microbial genomic DNA with the following universal primers: 338F (5′-ACTCCTACGGGAGGCAGCAG-3′) and 806R (5′-GGACTACHVGGGTWTCTAAT-3′). The PCR reactions were run on an ABI GeneAmp^®^ 9,700 (ABI, USA) with the following conditions: 95°C for 3 min, followed by 35 cycles of 95°C for 30 s, 55°C for 30 s, and 72°C for 45 s, and finally 72°C for 10 min. Three biological replicates of each sampling site were performed.

High-throughput sequencing on the Illumina MiSeq 2,500 platform at Majorbio Biotechnology Company (Beijing, China) was conducted for the V3–V4 region of the bacterial 16S rRNA gene. The sequencing depth of 16S rRNA sequencing was determined by generating an average of 48,296 sequences per sample, for a total of 579,550 sequences. Raw 16S rRNA gene sequencing reads were processed as follows: initially, raw reads underwent demultiplexing and quality filtering using fastp (version 0.20.0) (Chen et al., 2018). Subsequently, the reads were merged employing FLASH (version 1.2.7) (Magoč and Salzberg, 2011), adhering to the subsequent criteria: (1) Truncation of 300-bp reads transpired at any point with an average quality score of less than 20 over a 50-bp sliding window; truncated reads shorter than 50 bp and those containing ambiguous characters were discarded. (2) Assembly relied on sequences with overlapping regions longer than 10 bp, with a maximum mismatch ratio of 0.2 in the overlap region; unassembleable reads were discarded. (3) Samples were distinguished based on barcode and primers, with sequence direction adjusted. Exact barcode matching was required, with a tolerance of two nucleotide mismatches in primer matching. Sequences with ≥97% similarity were assigned to the same operational taxonomic units (OTUs), which were subsequently clustered utilizing UPARSE (version 7.1) (Edgar, 2013), followed by the identification and removal of chimeric sequences. Taxonomic classification of each representative OTU sequence was performed using the RDP Classifier (version 2.2) (Wang et al., 2007) against the 16S rRNA database (version 138), with a confidence threshold set at 70%. The datasets for this study can be found in the NCBI SRA repository, accession numbers PRJNA1096924.

### 2.7 Data analysis

The effective tags were classified against the SILVA SSU rRNA database (version 138) (Quast et al., 2012), and species annotation was analyzed using the mothur method (Kozich et al., 2013). Alpha indexes (Chao and Shannon) were calculated prior to rarefaction using the QIIME software (version 1.9.1) (Caporaso et al., 2010). To compare and analyze the differences in the dominant microbial species in different sediment samples, the heatmap was developed using the pheatmap software package in R software (version 4.3.1). The R package vegan was utilized for analyzing microbial structure differences between sites, encompassing principal coordinate analysis (PCoA) and redundancy analysis (RDA), with difference statistics computed using the *anosim*() function (Oksanen et al., 2015).

The correlations between environmental parameters and microbes were analyzed using Spearman’s correlation analysis with the criteria *p* < 0.05 and r > |0.8| with R software (4.3.1), and then the network plot was visualized using Gephi software (version 0.10.1, WebAtlas, France).

BugBase, PICRUSt2, and Functional Annotation of Prokaryotic Taxa (FAPROTAX) were used to predict putative functions of sediment microbes from taxonomic genes through analyses of the 16S rRNA gene. BugBase^1^ was used to determine the high-level seven phenotypes present in microbiome samples (Ward et al., 2017). These include gram positive, gram negative, biofilm forming, pathogenic, mobile element containing, oxygen utilizing (including aerobic, anaerobic, and facultatively anaerobic), and oxidative stress tolerant. PICRUSt2 (version 2.2.0) uses marker gene data and databases such as KEGG to predict putative microbial metabolic functions (Douglas et al., 2020). FAPROTAX (version 1.2.1) annotates taxa based on metabolic functions and ecological roles, particularly in nutrient cycling (Sansupa et al., 2021). Then, the Kruskal–Wallis rank sum test (Kruskal–Wallis *H* test) was used to analyze the differences in domain bacteria among sites. All statistical analyses and diagrams, including chart drawing and variance and correlation analysis, were conducted using R software (version 4.3.1).

## 3 Results

### 3.1 Physicochemical characteristics of surface water and sediment samples

**Figure 2:**
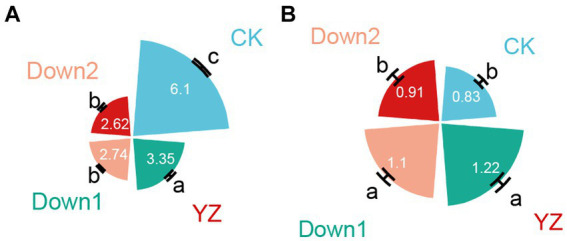
Dissolved oxygen (DO, mg/L) in overlying water **(A)** and sediment oxygen demand (SOD, g m^−2^ d^−1^) in sediment **(B)** samples.

The highest DO concentration, recorded at the CK site, was 6.11 mg/L, whereas a significant decrease in DO was observed from the YZ site (3.35 mg/L) to its lowest level at the Down2 site (2.62 mg/L) (Figure 2A). In contrast, downstream SOD exhibited an inverse trend compared to upstream. The average SOD values across all sites ranged from 0.83 to 1.22 (g m^−2^ d^−1^), with the *DO*-*t* standard curve presented in Supplementary Table S2. Compared to CK, the highest SOD was observed at YZ (1.22 g m^−2^ d^−1^), followed by the Down1 and Down2 sites (Figure 2B).

The physicochemical characteristics of surface water and sediment samples were monitored at various sites along the Gan River (Supplementary Figure S1), and establishing correlations between them was also accomplished (Supplementary Figure S2). TOC, TN, and NH_4_^+^-N concentrations in water and TOC, TN, and NH_4_^+^-N concentrations in sediments were significantly lower in DO depletion sites (YZ, Down1, and Down2) compared to the upstream control site (CK). These parameters exhibited a positive correlation with DO concentration (*p* < 0.05). Conversely, other physicochemical characteristics in overlying water (COD and NH_4_^+^-N) and sediment (TP) exhibited opposite trends. Additionally, YZ and Down1 had significantly lower sulfide concentrations compared to other sites (*p* < 0.05). These results indicate that TN and NH_4_^+^-N in water, as well as sulfide and TOC in sediment, might be the more important contributors to SOD (Jaiswal and Pandey, 2019).

### 3.2 Alpha diversity and principal coordinate analysis (PCoA)

**Figure 3:**
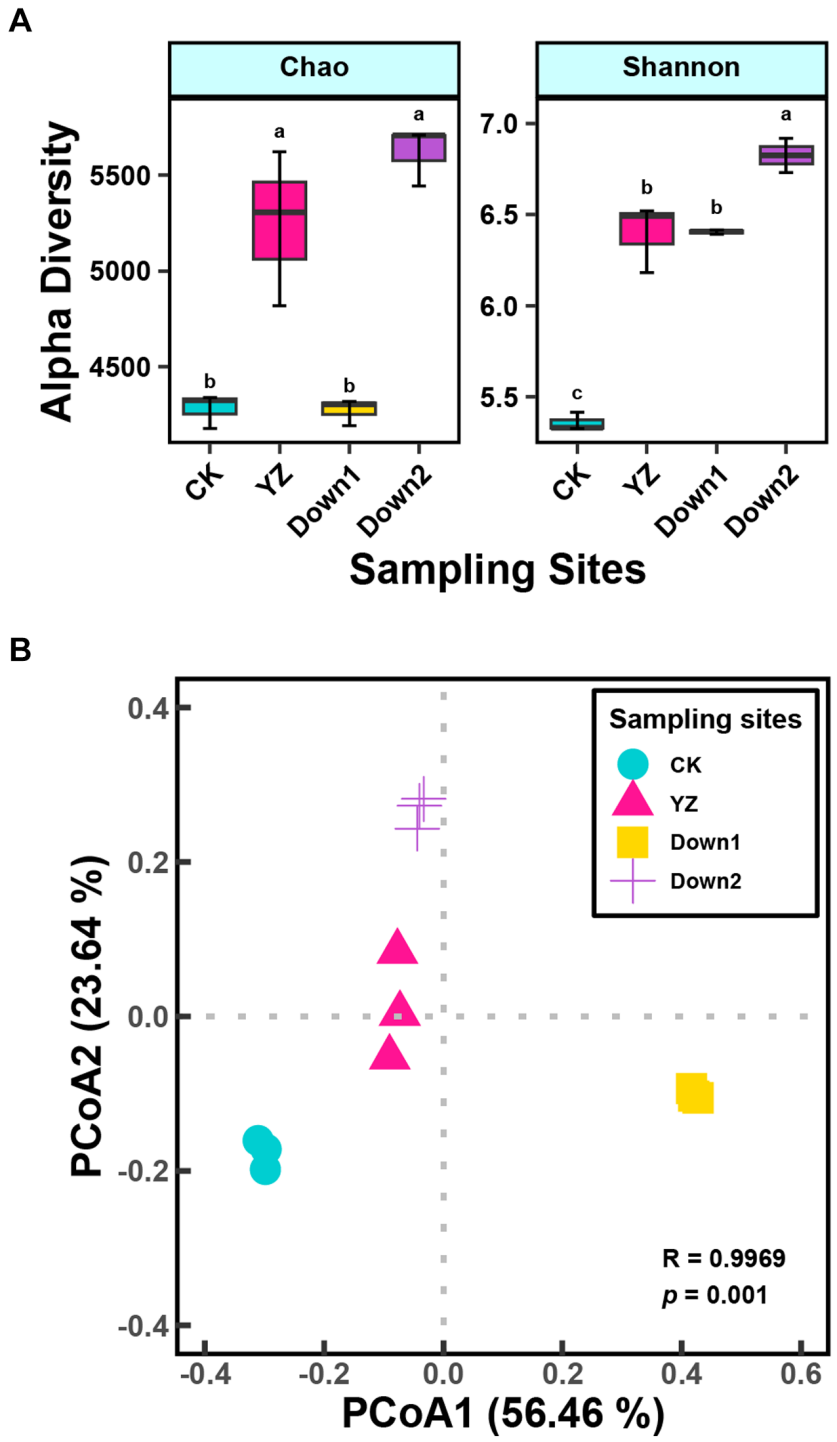
Microbial α-diversity index **(A)** and principal coordinate analysis (PCoA) of different sites on microbial composition based on the Bray–Curtis dissimilarity and ANOSIM analysis **(B)** in sediment along the Gan River.

To investigate the similarity of microbial community structure among four sites, alpha diversity was conducted (Figure 3A). The Chao index, primarily utilized to estimate the total observed species, showed lower values at the CK and Down1 sites compared to the YZ and Down2 sites. Additionally, the Shannon indexes at CK sites were significantly lower compared to those at DO depletion sites (YZ, Down1, and Down2).

PCoA was employed to assess the geographic clustering of microbial structure (Figure 3B). The first two components explained 56.46 and 23.64% of the total variance, respectively. The microbial structure exhibited significant differences (*p* < 0.001) among the four sites, with the DO depletion sites (YZ, Down1, and Down2) notably distinct from the CK site, which is positioned on the opposite side of the PC1 axis relative to the DO depletion sites.

### 3.3 Microbial community composition

**Figure 4:**
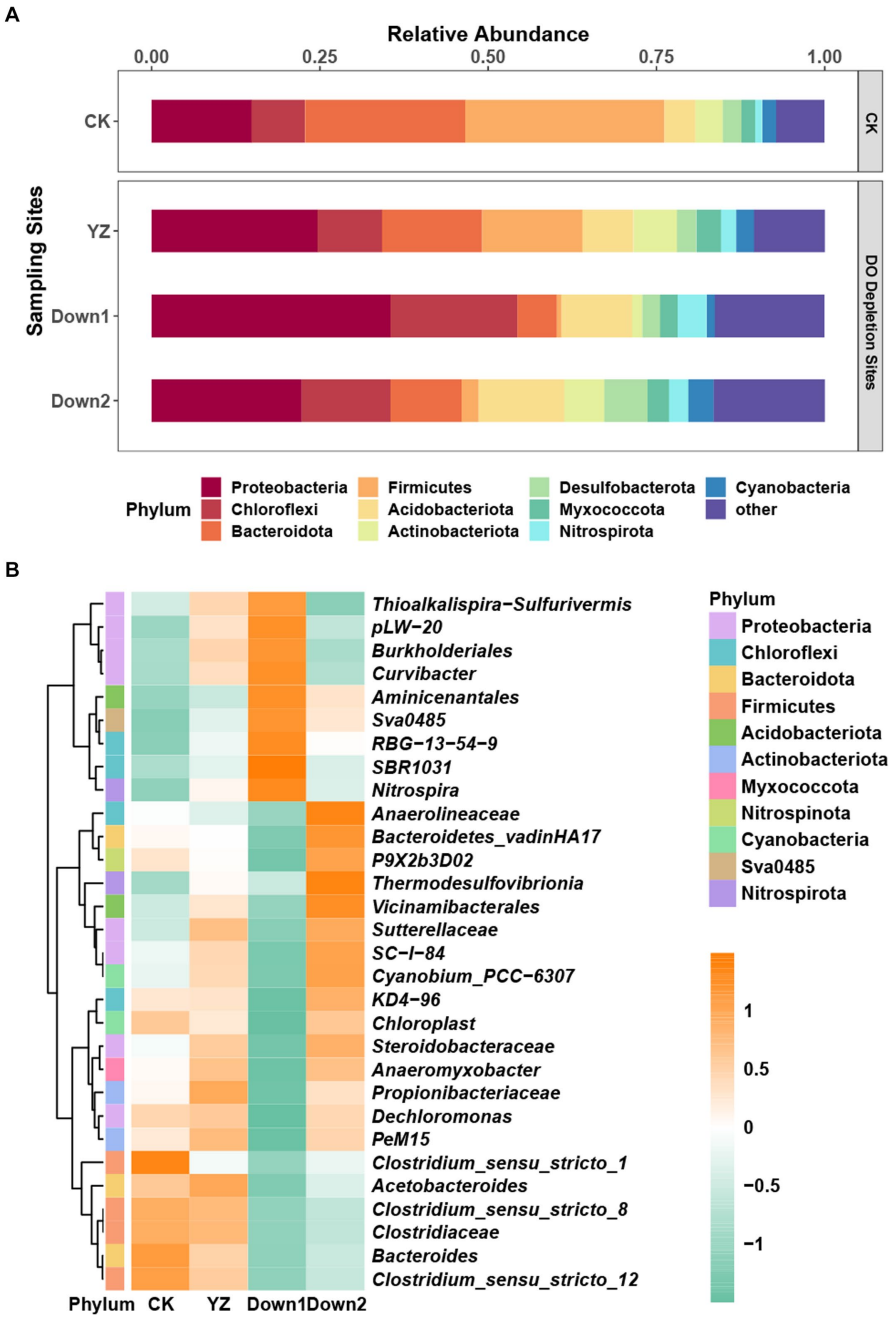
The relative abundance of the predominant microbial community in sediments: top 10 at the phylum level **(A)** and top 30 at the genus level **(B)**. The genus data were normalized using a log transformation. Numbers 1 to −1 on the scale bar represent the number of standard deviations from the mean *z*-score.

16S rRNA gene sequences assigned to bacteria span 64 phyla, 189 classes, 429 orders, 662 families, and 1,104 genera. To comprehensively understand the microbial community composition at various sediment sites along the Gan River, we analyzed the relative abundance at the phylum and genus levels (Figure 4). The core community at phyla level in sediment samples is depicted in Figure 4A, with Proteobacteria (14.85–35.49%) prevailing in most samples, followed by Bacteroidota (5.88–23.83%), Chloroflexi (7.93–18.87%), and Firmicutes (0.71–29.58%). Compared to the CK site, the DO depletion sites (YZ, Down1, and Down2) exhibited a significant increase in the relative abundance of Proteobacteria, Actinobacteria, and Nitrospirota, while Firmicutes and Bacteroidota displayed an opposite trend.

At the genus level, the relative abundance of the top 30 genera demonstrates significant variation across four different sites along the Gan River, as illustrated by a heatmap (Figure 4B). Dominant genera included *Anaerolineaceae* (3.82% in CK, 3.55% in YZ, 2.98% in Down1, and 5.48% in Down2), *Steroidobacteraceae* (3.00% in YZ and 3.68% in Down2), *Clostridium_sensu_stricto_1* (7.01% in CK), *Burkholderiales* (5.91% in Down1), and *SBR1031* (4.16% in Down1). The composition of dominant microorganisms at the genus level within the same phylum exhibited varying trends, indicating that the data at the genus level were more intricate than at the phylum level.

### 3.4 Differential OTUs

**Figure 5:**
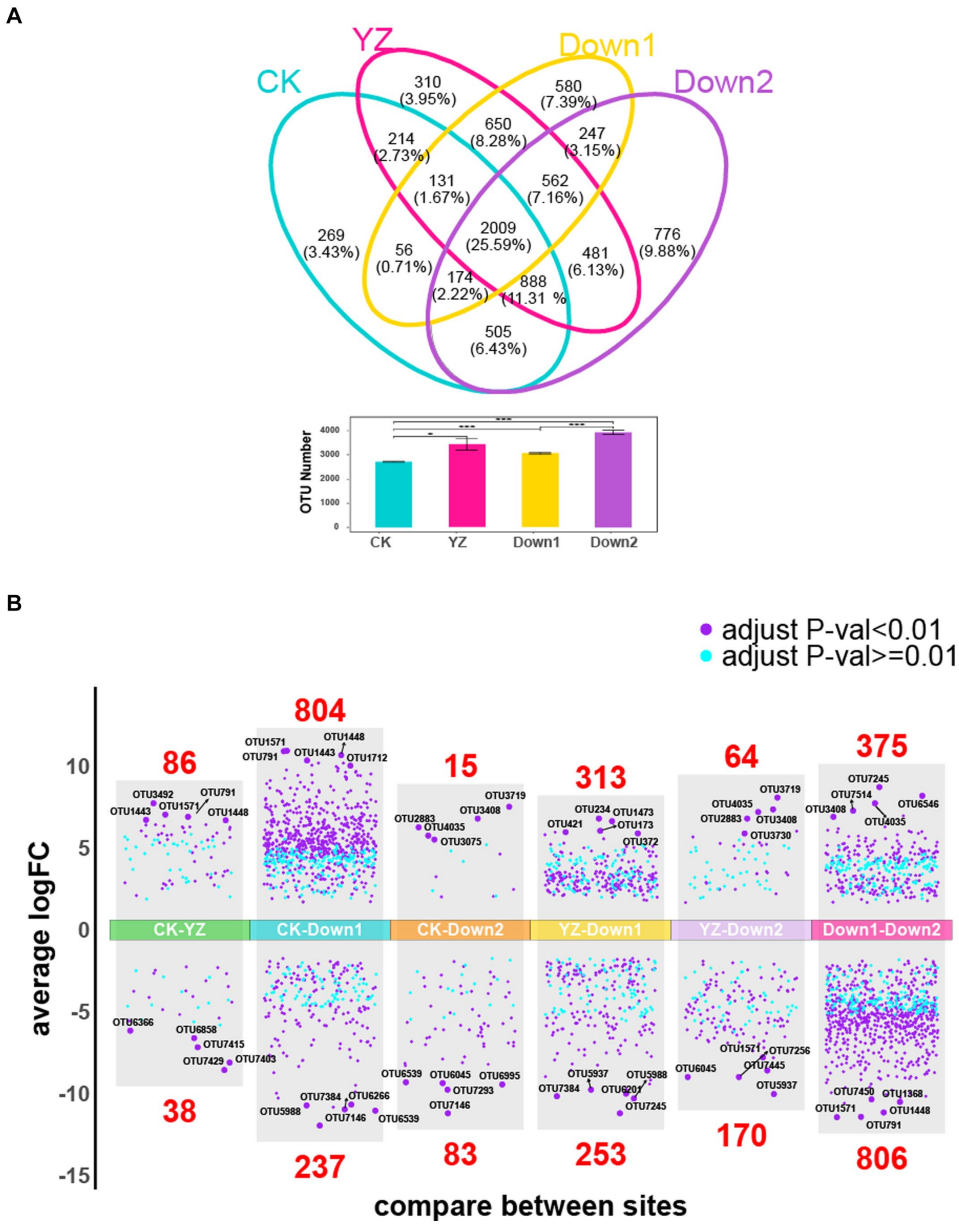
Differential OTUs along the Gan River. **(A)** Shared and unique OTUs within four sites analyzed by the Venn plot. The number and percentage represent the shared and unique OTUs. **(B)** Overall distribution of differential OTUs. The differential OTUs analyzed by volcano plot between YZ and other sites, respectively; an adjusted *p*-value <0.01 is indicated in purple, while an adjusted *p*-value ≥0.01 is indicated in blue; the number of differential OTUs between two sites is marked in red.

To further investigate the differences in microbial composition among all sediment samples, shared and unique OTUs were visualized using Venn diagrams (Figure 5A). The total number of OTUs at the CK site was significantly lower compared to those observed in DO depletion sites (YZ, Down1, and Down2). Among all four sites, 2,009 OTUs (25.59%) were shared, with Down2 having the highest count of unique OTUs at 776 (9.88%), followed by Down1 (7.39%), YZ (3.95%), and CK (3.43%). Volcano plot analysis was conducted to reveal the overall distribution of differential OTUs between CK and DO depletion sites (YZ, Down1, and Down2) (Figure 5B). The differential OTUs between each pair of sites were selected based on the criteria of log2|Fold-change| ≥ 2 and *p* < 0.05. The analysis revealed that 86, 804, and 15 OTUs were significantly increased in YZ, Down1, and Down2 sites, respectively, while 38, 237, and 83 OTUs were significantly decreased compared to CK. The top 5 OTUs showing significant increases or decreases in each pairwise comparison are listed in Supplementary Table S3. Notably, the abundance of OTU1571 (*SBR1031*), OTU791 (*Microscillaceae*), OTU1448 (*MND1*), OTU1443 (*Sulfurifustis*), OTU1368 (*DTB120*), and OTU7450 (*Burkholderiales*) was highest in Down1 sites, followed by the YZ site. Conversely, the abundance of OTU7146 (*AP-FeEnrich3*), OTU6266 (*Clostridium_sensu_stricto_12*), OTU7384 (*Bacteroides*), OTU6539 (*Caproiciproducens*), OTU5988 (*Sedimentibacter*), OTU6201 (*Paludicola_psychrotolerans*), OTU7429 (*Clostridium_sensu_stricto_16*), and OTU7293 (*Hydrogenoanaerobacterium_saccharovorans*) significantly decreased from upstream CK site to DO depletion sites (YZ, Down1, and Down2), with the lowest abundance observed at Down1.

### 3.5 Correlation between sediment microbial community structure and physicochemical characteristics of aquatic ecosystems

**Figure 6:**
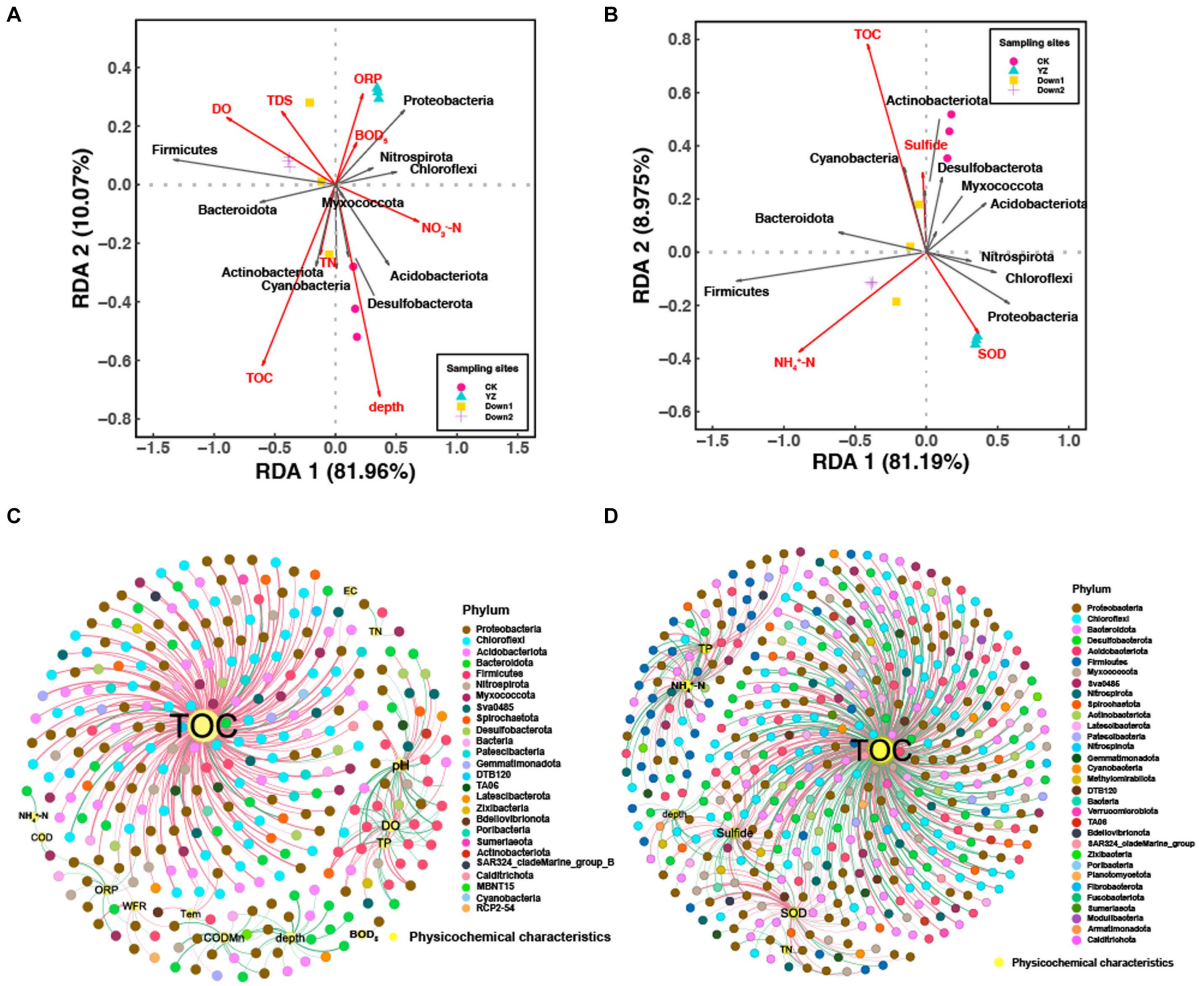
Redundancy analysis (RDA) of sediment microbial communities at the phylum level with environmental factors of water **(A)** and sediment **(B)**, respectively. Network analysis of sediment microbial communities with environmental factors of water **(C)** and sediment **(D)**. The criteria were *p* < 0.05 and r > |0.6|; red and green lines represent the positive and negative correlations between microbes and environmental factors, respectively; the thickness of the edges is proportional to the strength of the correlation; the color of nodes represents the microbes at the phylum level.

Redundancy analysis (RDA) was conducted to explore correlations between sediment microbial structure and water environmental factors (Figure 6A), as well as sediment microbial structure and sediment environmental factors (Figure 6B). RDA1 explained 81.96 and 81.19% of the total variance, while RDA2 explained 10.07 and 8.975%, respectively. This suggests that the variation in sediment microbial community composition at the phylum level can be well explained by the selected physicochemical characteristics in both overlying water and sediment. Whether in the overlying water or sediment, TOC consistently emerged as the most influential environmental factor on microbial structure, followed by water depth and DO, as well as NH_4_^+^-N and SOD in the sediment.

The microbial community composition in the sediments of the CK site was primarily influenced by TOC and the depth of overlying water, as well as TOC and sulfide in the sediments. Conversely, the microbial community composition in sediments of DO depletion sites (YZ, Down1, and Down2) was mainly driven by DO, TDS, and ORP in overlying water, NH_4_^+^-N, and SOD in sediments. At the phylum level, Firmicutes showed a positive correlation with DO (*p* < 0.05) (Figure 6A), while Proteobacteria exhibited a positive correlation with SOD (*p* < 0.05) (Figure 6B).

Co-occurrence network analysis provides important evidence regarding the relationship between OTUs and environmental variables both in water (Figure 6C) and sediment (Figure 6D). To better recognize the complex patterns of interrelationships among nodes, the nodes and edges were calculated as having significant topological properties where each node represents an environmental factor or OTUs, while each red or green edge represents positive and negative correlation, respectively. The network of OTUs and environmental factors in water comprised 326 nodes and 366 edges, whereas the network in sediment exhibited a significantly higher number of 502 nodes and 621 edges (Supplementary Table S4). Specifically, the nodes were predominantly distributed among 26 and 32 phyla, respectively. Most nodes were associated with TOC of water, accounting for 58.47%, while only 7.92% were related to DO (Figure 6C and Supplementary Table S5). Similarly, most nodes were associated with TOC of sediment, accounting for 59.42%, while only 9.98% were related to SOD (with 80.65% positive correlations and 19.35% negative correlations) (Figure 6D and Supplementary Table S6). These physicochemical characteristics mentioned above were considered the main factors determining the differences in the distribution of sediment bacterial communities in the Gan River, especially the concentration of TOC, whether in sediment or overlying water.

### 3.6 BugBase for predicting microbial phenotypes

**Figure 7:**
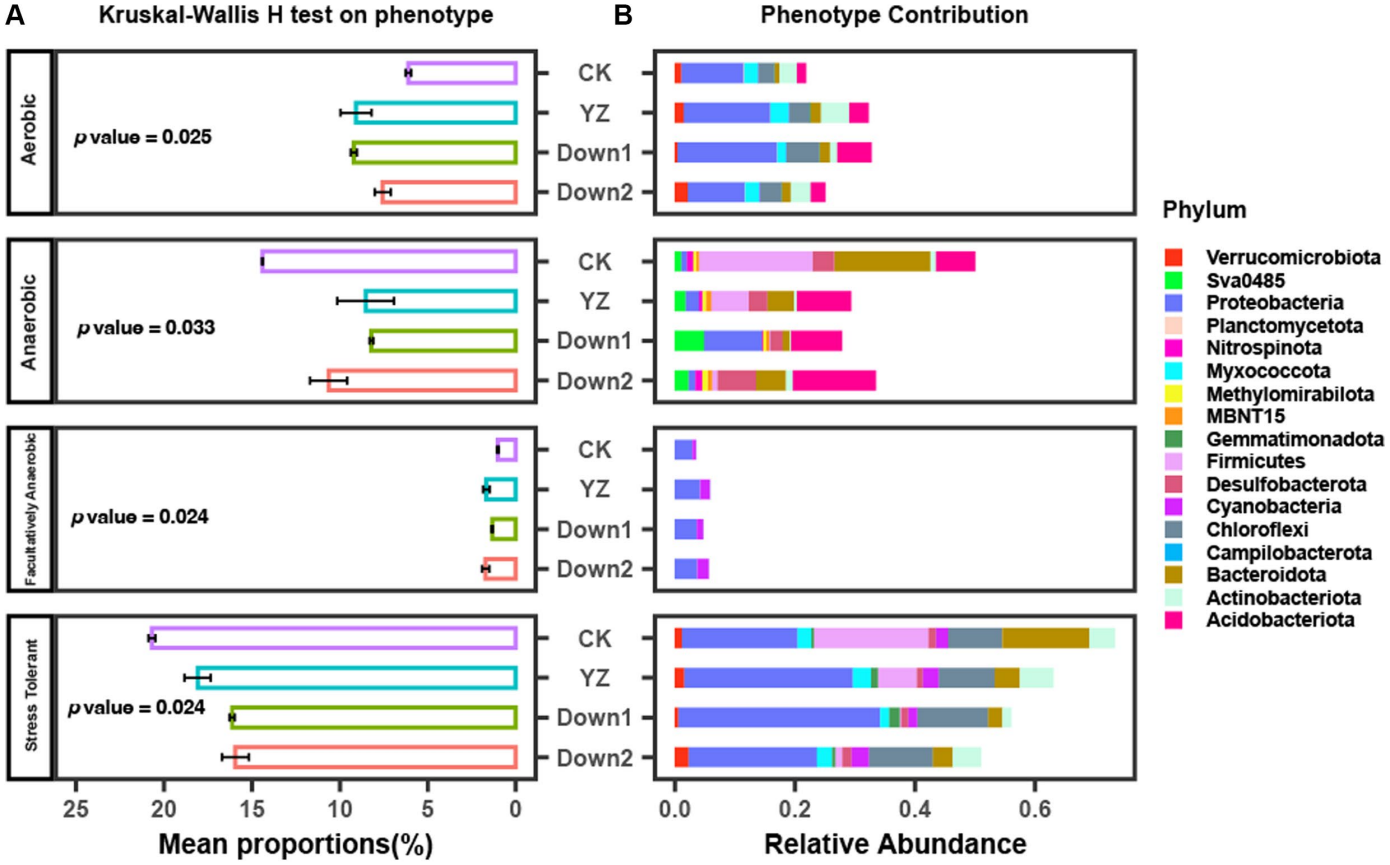
Phenotype of microbial communities predicted by using BugBase. **(A)** The mean proportions of phenotype and Kruskal–Wallis *H* test analyzed on phenotype. **(B)** The relative abundance of phenotype contribution at the phylum level.

The mean proportions of Oxygen Utilizing and Oxidative Stress Tolerant are shown in Figure 7 and Supplementary Figure S3. The results indicate that the mean proportions of Aerobic bacteria were significantly higher at DO depletion sites (YZ, Down1, and Down2) than those at the CK site, peaking at the YZ site, suggesting greater DO consumption at DO depletion sites. Similarly, the abundance of facultatively anaerobic bacteria showed similar trends to aerobic bacteria. However, the abundance of anaerobic and oxidative Stress tolerant bacteria exhibited contrasting trends to aerobic bacteria, with the highest abundance observed at the CK site. To predict the contribution of bacteria phyla to phenotype, the relative abundance of the top 10 phyla for each phenotype was also analyzed (Figure 7). Proteobacteria, as the dominant phylum, made the greatest contribution to aerobic bacteria, followed by Acidobacteriota, both exhibiting the highest abundance at the Down1 site.

### 3.7 Putative metabolic functions of sediment microbes’ prediction by PICRUSt2

**Figure 8:**
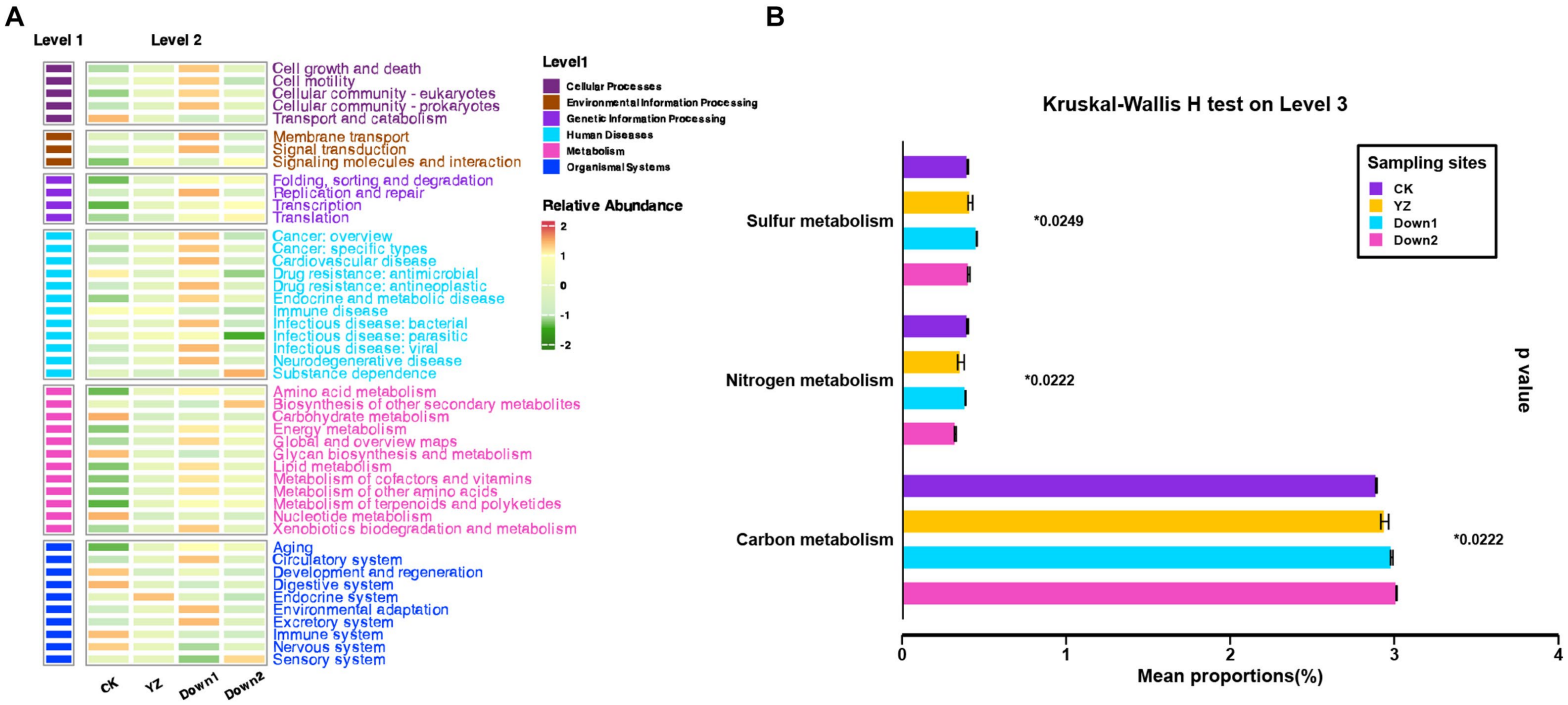
Functional categories (KEGG) of bacterial communities using PICRUSt2 analysis. **(A)** The heatmap of relative abundance on pathway level 2. **(B)** Kruskal–Wallis *H* test on pathway level 3.

The putative metabolic functions of microbes were predicted using PICRUSt2 analysis and classified into three levels based on the Kyoto Encyclopedia of Genes and Genomes (KEGG) database, aiming to discern variations in microbial community function under different physicochemical characteristics (Figure 8). The results showed that the average percentage of metabolism (77.19%) was the highest among the other five categories at the first level, followed by genetic information processing (7.66%), environmental information processing (5.15%), cellular processes (4.59%), human diseases (3.56%), and organismal systems (1.85%) (Supplementary Figure S4A). The level of metabolism was roughly the same at CK, YZ, Down1, and Down2 sites. At the secondary level, the gene copy number of all 46 sub-functions was shown in a heatmap (Figure 8A). The main functional genes were involved in the following pathways: carbohydrate metabolism (9.0%), amino acid metabolism (7.4%), energy metabolism (4.8%), metabolism of cofactors and vitamins (4.4%), and translation (3.3%) (Supplementary Figure S4B). Carbohydrate metabolism was significantly enriched at CK, while amino acid metabolism and energy metabolism exhibited the opposite trends and were enriched at DO depletion sites (YZ, Down1, and Down2). Thus, the mean proportion of functional genes for carbon, nitrogen, and sulfur metabolism was performed at the tertiary level (Figure 8B). The relative abundance of carbon metabolism gradually increased from the CK site to DO depletion sites (YZ, Down1, and Down2), consistent with the TOC contents along the river. The mean proportion of genes related to sulfur metabolism was higher at YZ and Down1 sites, responding to the lower sulfide concentration found in the sediment at these sites.

### 3.8 The prediction of putative ecological functions of sediment microbes by FAPROTAX

**Figure 9:**
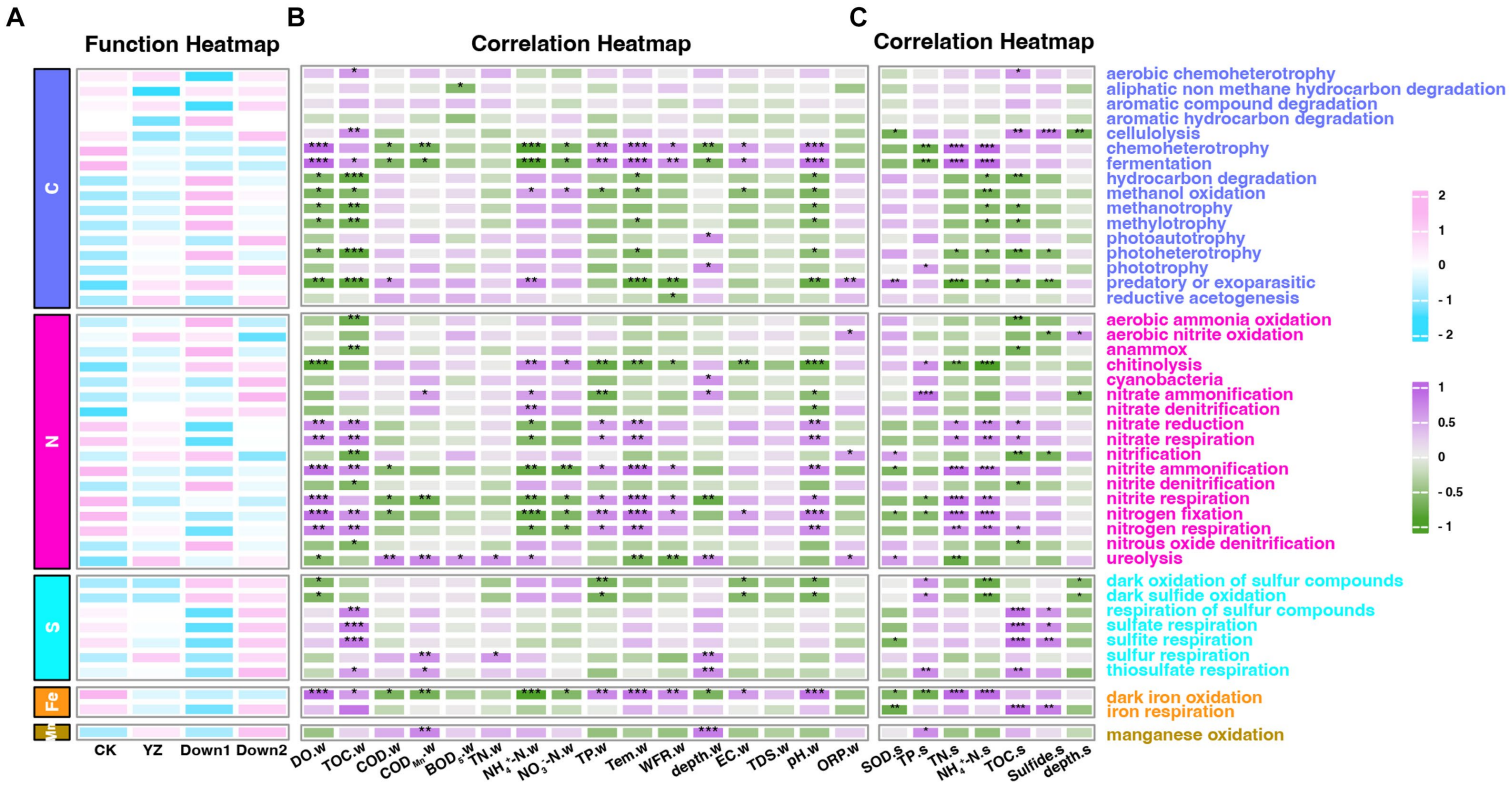
The ecological functions of sediment bacteria predicted by FAPROTAX. **(A)** The abundance of ecological functional groups of bacteria. **(B)** The correlation heatmap of the abundance of functional microbial groups and water physicochemical parameters. **(C)** The correlation heatmap of the abundance of functional microbial groups and sediment physicochemical parameters. Numbers 2 to −2 on the scale bar represent the number of standard deviations from the scale. Number of scale bars: 1 (purple), positive relationship; −1 (green), negative relationship. “*” represents the degree of significance: *p < 0.05; **p < 0.01; ***p < 0.001.

The relative abundance of putative ecological functions of sediment microbes was investigated using FAPROTAX (Figure 9A), while also exploring the potential interactions between microbial processes and environmental factors in both overlying water (Figure 9B) and sediment (Figure 9C). The results revealed that 1,107 OTUs of 8,504 bacteria (13.02%) were assigned to at least one functional group, and OTUs with a value of ≥1 were assigned to 64 distinct functional groups. The dominant functional groups of predicted bacteria were involved in carbon (C) cycling (46.45–66.66%), nitrogen (N) cycling (7.42–15.37%), and sulfur (S) cycling (1.34–2.71%), while only a few groups were involved in iron (Fe) cycling (0.07–1.42%) and manganese (Mn) cycling (0.28–0.68%) (Figure 9A).

The DO depletion sites exhibited higher levels than those in the CK site across 10 functional groups (carbon cycle: hydrocarbon degradation, methanol oxidation, methanotrophy, methylotrophy, photoheterotrophy and predatory or exoparasitic; nitrogen cycle: chitinolysis and ureolysis; sulfur cycle: oxidation of sulfur compounds and sulfide oxidation), which were negatively correlated with the concentration of DO (*p* < 0.05). Conversely, the remaining nine functional groups (Carbon cycle: chemoheterotrophy and fermentation; Nitrogen cycle: nitrate reduction, nitrate respiration, nitrite ammonification, nitrite respiration, nitrogen fixation, and nitrogen respiration; Fe cycle: iron oxidation) displayed contrasting trends and were enriched at the CK site. The SOD exhibited a positive correlation with three functional groups (Carbon cycle: predatory or exoparasitic; Nitrogen cycle: nitrification and ureolysis), while six groups (Carbon cycle: cellulolysis; Nitrogen cycle: nitrite ammonification and nitrogen fixation; Sulfur cycle: sulfite respiration; Fe cycle: iron oxidation and iron respiration) demonstrated an inverse relationship.

## 4 Discussion

### 4.1 Relationship between DO, SOD, and physicochemical characteristics of aquatic ecosystem

SOD is influenced by various environmental factors, including both physical and chemical parameters in water and sediment (Lee et al., 2018). The role of DO in regulating SOD varies, with lower levels of DO playing a crucial role (Lehrter et al., 2012), while its influence diminishes at higher levels (Zhang et al., 2017). In our study, DO levels in depletion sites ranged from 2.62 to 3.35 mg/L, potentially limiting SOD, as supported by previous research (Chi et al., 2021). However, conflicting findings suggest that DO may not always be the determining factor for SOD (Zhang et al., 2017).

DO depletion can result from factors such as increasing temperature, decreasing flow, and elevated organic carbon input into riverine ecosystems (Jaiswal and Pandey, 2019). SOD is typically promoted at higher temperatures ranging from 10 to 30°C, exhibiting a close relationship with temperature (Chi et al., 2021). In our study, water temperature ranged from 33.4 to 33.7°C in depletion sites, showing a negative correlation with SOD (*p* < 0.05). It is assumed that temperature may limit SOD when it exceeds 30°C. Water flow rate (WFR) affects oxygen flux into river water, influencing sediment oxygen concentration and regulating SOD (Jaiswal and Pandey, 2019). Diminished WFR exacerbates pollutant accumulation in sediment, contributing to elevated respiration rates and oxygen-demanding chemical concentrations during the summer, thus increasing SOD and reducing DO levels (Jaiswal and Pandey, 2019). Our study found higher WFR in control sites compared to depletion sites, possibly due to backwater influence from downstream Futou Lake. Furthermore, we observed a positive relationship between DO and pH (Supplementary Figure S2). A previous study also found that in 2005 and 2006, when pH dropped to approximately 7.2 in the upstream zone, DO levels fell below 2 mg/L, while in 2003, when water pH exceeded 8.4 in the surface water of the east and west shelves, DO rose above 10 mg/L due to the synergistic effect of phytoplankton photosynthesis and carbon system regulation (Li et al., 2020).

The concentration of NO_3_^−^-N in water showed a negative correlation with DO concentration (Supplementary Figure S2) and had an insignificant correlation with the abundance of denitrification (nitrite and nitrate) functional groups (Figure 9). This aligns with findings suggesting denitrification is regulated more by fungal and chemodenitrification processes than bacteria with increasing nitrate concentrations (0.5–2.0 mg/L) (Li et al., 2023). Chemodenitrification is an abiotic process involving iron oxidation coupled with nitrate/nitrite reduction under anoxic and high nitrate conditions (Li et al., 2023). This process aligns with our findings, which indicate a negative correlation between nitrate concentration and the abundance of functional groups involved in iron oxidation (*p* < 0.05) and nitrite reduction (*p* > 0.05) (Figure 9). DO concentration negatively correlated with NH_4_^+^-N (*p* < 0.001) and COD (*p* < 0.05), consistent with previous studies (Zhang et al., 2022), while NH_4_^+^-N showed a positive relationship with SOD, indicating its role in promoting SOD.

Sediment and overlying TOC were both negatively correlated with SOD (Supplementary Figure S2), consistent with previous research suggesting a synchronous trend with SOD (Jaiswal and Pandey, 2019). Approximately 28% of SOD originates from sulfide mineralization (Barcelona, 1983), consistent with our findings showing a negative correlation between sediment sulfide and SOD. As sulfide mineralizes, its concentration decreases while SOD levels increase (Supplementary Figures S1, S2).

### 4.2 Interactions among DO, SOD, and sediment microbial community structure

In this study, we found a close interconnection between DO, SOD, and sediment microbial community structure. The sediment microbes exhibited distinct structures across varying physicochemical characteristics of surface water and sediment in the Gan River. Specifically, the DO concentration showed a negative correlation with SOD (Supplementary Figure S2). Previous investigations of estuaries with seasonal hypoxia revealed a negative correlation between microbial diversity and DO levels (Park et al., 2020). Consistent with these findings, we observed a negative association between DO concentration and both microbial diversity (Shannon index) and species richness (Chao index) (Figure 3).

At the phylum level (Figure 4A), Proteobacteria predominated in most samples, followed by Bacteroidota, Chloroflexi, and Firmicutes, which have been widely identified as the predominant bacteria in river sediment (Hou et al., 2021). The RDA analysis revealed that DO negatively correlated with the abundance of Proteobacteria (Ouyang et al., 2020) and positively correlated with Firmicutes and Bacteroidetes (Figure 6A). The TOC content was the primary factor driving the sediment microbial community, according to the RDA and co-occurrence network analysis (Figure 6), aligning with a previous study (Duan et al., 2022).

The variation in sediment microbial communities was also assessed at the genus level. *Nitrospira*, as an obligate chemolithoautotroph (Daims et al., 2016), exhibits a microaerobic lifestyle by utilizing formate and hydrogen as substrates for growth under aerobic conditions and reducing nitrate to nitrite under anaerobic conditions (Koch et al., 2019). This is consistent with the findings of our study, where *Nitrospira* dominated sediments in DO depletion sites (YZ, Down1, and Down2) (Figure 4B); network analysis revealed a negative correlation between *Nitrospira* and DO while showing a positive correlation with SOD (Figures 6C,D). Sulfur-oxidizing bacteria, such as *Sulfurifustis* and *Sulfuritalea*, can utilize oxygen and arsenate as alternative electron acceptors (Watanabe et al., 2017) and are responsible for consuming sulfide (Kojima et al., 2014). This metabolic adaptation leads to reduced levels of sulfide due to their enrichment at the YZ and Down1 sites (Supplementary Figure S1B), exhibiting a negative correlation with DO concentrations (Figure 6C). Within the Firmicutes phylum, the genus *Clostridium* has a strictly anaerobic lifestyle (Morvan et al., 2021), confirming the higher abundance of *Clostridium sensu stricto 1*, *Clostridium sensu stricto 8*, and *Clostridium sensu stricto 12* found in DO depletion sites (YZ, Down1, and Down2) and negatively correlated with DO in this study.

Co-occurrence network analysis revealed the differential distribution of OTUs among different sites, and the abundance of OTUs showed a strong correlation with DO and SOD (Figures 6C,D). The chemoheterotrophic *Bacteroides* generate energy through the fermentation of carbohydrates (Ismaeil et al., 2018). OTU7146 (*Bacteroides*) was enrichment in the CK site but absent in DO depletion sites (YZ, Down1, and Down2) (Figure 4B) and exhibited a significant negative correlation with DO, leading to a higher abundance of bacteria associated with fermentation in the CK site (Figure 9).

OTU1003 (*Curvibacter*) and OTU670 (*pLW-20*), belonging to Proteobacteria, exhibited enrichment in DO depletion sites (YZ, Down1, and Down2) (Figure 4B), showing a positive correlation with SOD (Figure 6D; Supplementary Table S6), indicating higher DO consumption in DO depletion sites. The *Curvibacter* genus is an aerobic chemoheterotrophic organism capable of utilizing oxygen to degrade organic contaminants, possibly relating to aerobic metabolic or co-metabolic degradation of chlorinated hydrocarbons (CHCs) (Li et al., 2022). The *pLW-20* belongs to the Methylomonadaceae family, a previous study revealed that CH_4_-oxidizing methanotrophs in the Methylomonadaceae family were identified in lake sediments (Deng et al., 2024), coinciding with a higher abundance of microbes associated with methanotrophy in DO depletion sites (YZ, Down1, and Down2) of the Gan River (Figure 9A).

Overall, the results suggest that the composition and diversity of sediment microbial communities varied in response to physicochemical parameters in the Gan River. Certain microorganisms enriched in DO depletion sites were found to be involved in aerobic metabolism by utilizing O_2_ as an electron acceptor, exhibiting a negative correlation with DO and a positive correlation with SOD.

### 4.3 Increased SOD due to DO consumption by the phenotype aerobic microbes

In this study, we focus on the composition and structure of sediment microbial communities under varying DO concentrations while also exploring their functional contributions to rising SOD and consumption of DO. The majority of prokaryotes rely on oxygen respiration, making oxygen indispensable for multicellular life as the terminal electron acceptor in aerobic respiration, which is widely acknowledged as the most efficient form of energy metabolism (Fenchel and Finlay, 2008).

The phenotype of oxygen utilizing (including aerobic, anaerobic, and facultatively anaerobic) and oxidative stress tolerant were predicted using BugBase (Figure 7). Theoretically speaking, Aerobic bacteria will thrive in the water–sediment interface where abundant DO supports their nutrient degradation, while the anaerobic bacteria will gradually replace aerobic microorganisms and become the dominant bacteria communities when the DO is depleted by aerobic processes (Broman et al., 2017). In this study, it was observed that the abundance of aerobic bacteria was higher in the DO depletion sites, and the predominant aerobic phenotype was characterized by the dominance of Proteobacteria, followed by Acidobacteriota and Chloroflexi (Figure 7). This phenomenon may be attributed to the aerobic bacteria consuming a large amount of oxygen and rapidly growing to become dominant bacterial species during the sampling period, while the abundance of anaerobic bacteria is relatively low at this time. Consequently, there was a decrease in oxygen levels in the overlying water and an increase in SOD rate, while anaerobic bacteria did not become predominant. The facultatively anaerobic bacteria can thrive in either oxic or anoxic conditions (Fu et al., 2015), and the presence of oxygen and other electron acceptors fundamentally influences both catabolic and anabolic pathways (Unden et al., 1994). The DO depletion sites (YZ, Down1, and Down2) exhibited a higher abundance of facultative anaerobic bacteria than those in the CK site (Figure 7); however, this phenotype displayed the lowest overall abundance among the seven phenotypes, suggesting relatively limited oxygen consumption and minimal impact on SOD rate.

### 4.4 DO depletion due to microbial metabolic processes

When aerobic microorganisms degrade nutrients on the surface of sediments (Emeis et al., 2000), these metabolic processes deplete available oxygen, resulting in anoxic conditions in both the sediment and the overlying water column (Middelburg and Meysman, 2007). Although the permeability of DO in sediments is limited, rendering its detection challenging in sediments, the profound biogeochemical responses of anoxic sediments to fluctuations in DO concentrations in the overlying water are frequently observed (Liu et al., 2021).

In all major pathways, the genes associated with metabolism ranked highest among the metabolic function categories on level 1 (Supplementary Figure S4A), as analyzed by PICRUSt2, indicating that bacterial communities possess a high metabolic potential (Liang et al., 2020). During metabolic processes, microbial communities in sediments exhibit stratification based on the availability of electron acceptors, which are utilized in order of decreasing energy gain: O_2_, NO_3_^−^, Mn^3+^, and Mn^4+^ oxides, Fe^3+^ oxides, and SO_4_^2−^ (Armstrong-Altrin et al., 2022). The utilization of alternative electron acceptors, in turn, impacts the oxygen reduction potential within the sediments (Broman et al., 2017). The microbial community of sediments in Gan River was predicted by FAPROTAX (Figure 8), due to its crucial roles in driving biogeochemical processes (Louca et al., 2016), such as the carbon cycle (Goetghebuer et al., 2017), nitrogen cycle (Boyer et al., 2006), sulfur cycle (Cai et al., 2019), iron cycle (Sun et al., 2021), and manganese cycle (Sutherland et al., 2018).

The metabolism of carbon and nitrogen is tightly coupled due to their status as the predominant nutrient elements for all living organisms (Zhang et al., 2018). The abundance of a large number of functional groups associated with C and N cycling exhibited significant variations across DO depletion sites (YZ, Down1, and Down2) and CK site (Figure 9A), implying that the alteration in microbial community composition would impact the cycling of carbon and nitrogen in sediments (Monteux et al., 2020).

A previous study revealed a significant decrease in carbon metabolic level when the DO content exceeded 4.5 mg/L (Wang et al., 2023), accompanied by a lower carbon metabolic level at the CK site with a DO concentration of 6.11 mg/L in the Gan River (Figure 8B). This decline can be attributed to alterations in microbial community diversity, symbiotic balance, functional genes, and metabolic processes caused by the elevated DO content, ultimately impacting carbon metabolism (Wang et al., 2023). Methylotrophy is defined as the capacity of microorganisms to utilize reduced one-carbon compounds, such as methane and methanol, as sole carbon and energy sources for growth (Gregory et al., 2022). Methane can be directly used for fermentation or first oxidized to methanol through biological or chemical means (Gregory et al., 2022). An essential characteristic of aerobic methanotrophs is the oxidation of methane to methanol using O_2_ (Catling et al., 2007). The methanol-utilizing bacteria can use methanol as a source of carbon and energy, converting it into CO_2_ (Kolb, 2009). The groups of methanol oxidation, methanotrophy, and methylotrophy exhibited higher abundance in the DO depletion sites (YZ, Down1, and Down2) compared to the CK site, showing a significant negative relationship with DO concentration (*p* < 0.05) (Figure 9), indicating these processes consume an amount of oxygen in the DO depletion sites in the Gan River. Recently, a previous study revealed that the Fermentation process was predominantly governed by Bacteroidota (Su et al., 2024). The presence of Fermentation bacteria exhibited a decline with decreased Bacteroidota abundance in DO depletion sites of the Gan River, indicating a reduction in carbon dioxide emissions (Liu et al., 2023).

The global nitrogen cycle plays a pivotal role in the biogeochemistry of the Earth (Fowler et al., 2013), encompassing distinct nitrogen transformation processes orchestrated by diverse microorganisms, including assimilation, ammonification, nitrification, denitrification, anaerobic ammonium oxidation (anammox), nitrogen fixation, and dissimilatory nitrite reduction to ammonium (Feng et al., 2023).

Nitrogen-fixing cyanobacteria possess distinct physiological characteristics that facilitate their dominance and the formation of blooms (Zhao et al., 2019). During these cyanobacterial blooms, the extensive proliferation and decomposition of algae result in a reduction in dissolved oxygen levels within the water (O’Boyle et al., 2016). Furthermore, cyanobacterial blooms enhance nitrification by providing NH_4_^+^-N and DO, as well as TN removal through nitrification–denitrification (Peng et al., 2017). In the Gan River, DO depletion sites exhibit a significantly higher abundance of cyanobacteria associated with the nitrogen cycle (Figure 9A). Additionally, there is a negative correlation between the abundance of nitrification and denitrification and the concentration of DO, which explains the increased oxygen consumption and enhanced TN removal observed at DO depletion sites (Supplementary Figure S1). The genus *Bacillus* and *Zoogloea* were enriched in DO depletion sites of the Gan River, which are responsible for nitrate denitrification and are facultative anaerobes, so oxygen does not inhibit their anaerobic metabolism.

Microbial sulfur metabolisms play a crucial role in driving the biogeochemical sulfur cycle over geological timescales and coupling it to the carbon, oxygen, and iron cycles (Fike et al., 2015). The reduction of sulfate to hydrogen sulfide is a common phenomenon in anaerobic sediments caused by sulfate-reducing bacteria, and the generated sulfides can be re-oxidized to sulfates under aerobic conditions (Broman et al., 2017). In this study, the proportion of sulfur metabolism was higher in DO depletion sites compared to the CK site (Figure 8B), and there was a significant negative correlation between groups involved in sulfide oxidation and sulfur compound oxidation with DO concentration (*p* < 0.05) in the Gan River (Figure 9C). Importantly, *Sulfurospirillum*, *Thioploca*, and *Thiobacillus* genera were found to be enriched in DO depletion sites within the Gan River, indicating their active participation in sulfur oxidation processes.

The oxidation of manganese serves as a metabolic pathway for microorganisms to acquire energy and support the growth of chemolithoautotrophic microbes (Yu and Leadbetter, 2020). Interestingly, the abundance of bacteria associated with manganese oxidation was higher in DO depletion sites compared to those in the CK site and exhibited a negative correlation with DO levels, suggesting that this process also contributed to DO consumption and promotes the SOD rate in DO depletion sites of the Gan River.

Furthermore, we have also observed that a multitude of functional groups (such as dark iron oxidation and iron respiration) associated with iron cycling exhibit alterations in response to varying DO conditions (Figure 9C). The phenomenon of rapid oxidation of Fe(II) in the subsurface environment by O_2_ under neutral pH has been extensively documented (Huang et al., 2021), confirming a significant positive correlation between iron oxidation and DO concentration (*p* < 0.001), as well as a negative correlation with SOD (*p* < 0.05) in Gan River (Figures 9B,C). A higher abundance of bacteria associated with iron oxidation was observed in the CK site compared to those in DO depletion sites of the Gan River (Figure 9A), particularly with an increased prevalence of *Gallionella* and *Azospira* genera involved in this biogeochemical process.

The study found that in DO depletion sites, sediment microorganisms engaged in aerobic metabolic processes, leading to oxygen consumption and an increase in SOD. It is speculated that eliminating indigenous microorganisms associated with toxic H_2_S and CH_4_ may contribute to the reoxygenation of water bodies, aligning with similar results reported in the literature (Broman et al., 2017). Identifying and controlling factors contributing to DO consumption will aid in restoring dissolved oxygen levels in the Gan River water bodies and provide valuable information for their management and conservation efforts. These findings pertaining to metabolism underscore the pivotal role of these processes in facilitating DO consumption and promoting the rate of SOD at DO depletion sites of the Gan River.

## 5 Conclusion

**Figure 10:**
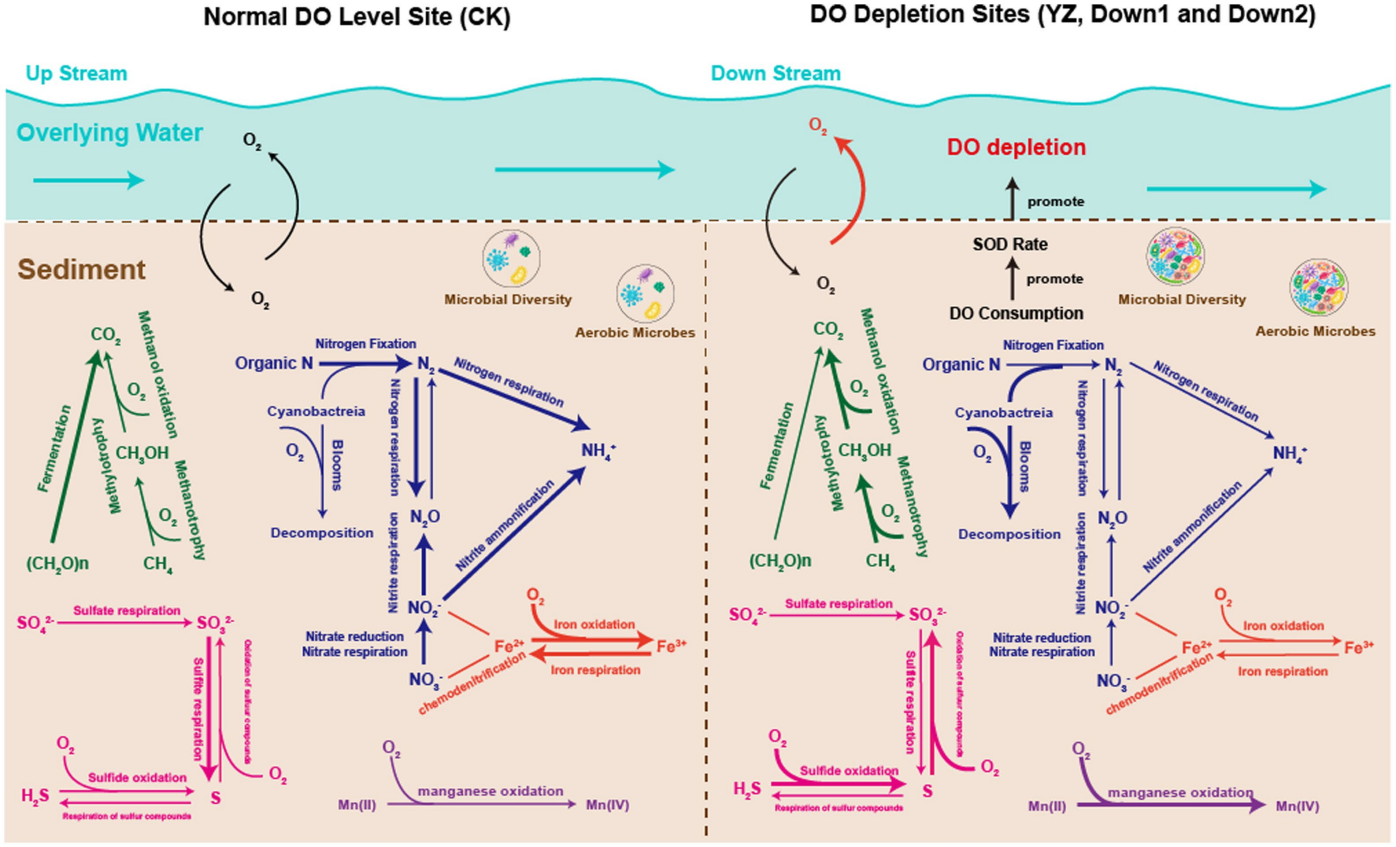
Overview of the biogeochemical process in the sediments of the Gan River. C cycle is shown in green; N cycle is shown in blue; S cycle is shown in pink; Fe cycle is shown in red; and Mn cycle is shown in purple. The thickness of the line corresponds to the abundance of functional microbial groups.

In conclusion, this study investigated the impact of the relationship between the putative functions of sediment microbes and SOD on DO depletion in the Gan River. The richness (Chao) and diversity (Shannon) of sediment microbes showed a negative correlation with DO concentration. Moreover, the sediment microbial structure differed between DO depletion sites and CK sites, with Proteobacteria predominating at the phylum level across all sampling sites. TOC emerged as the primary factor shaping sediment microbial structure, as evidenced by redundancy analysis (RDA) and co-occurrence network analysis. The SOD levels were notably affected by water temperature, flow rate, and organic matter in the aquatic environment, particularly by the putative metabolic and ecological functions of sediment microbes predicted using BugBase, PICRUSt2, and FAPROTAX software. The relationship between metabolic pathways and DO concentration is illustrated in Figure 10. Aerobic phenotype microbes consume a significant amount of oxygen during metabolic processes involving carbon, nitrogen, and sulfur, becoming dominant in DO depletion sites and consequently increasing SOD rates. Metabolic and ecological pathways related to the carbon cycle (methanol oxidation, methanotrophy, and methylotrophy), sulfur cycle (sulfide oxidation and sulfur compound oxidation), manganese cycle (manganese oxidation), and nitrogen cycle (Cyanobacteria, nitrification, and denitrification) were notably heightened at DO depletion sites compared to CK sites, displaying a significant negative correlation with DO concentration (*p* < 0.05) (Figure 9). Furthermore, the study underscores that reoxygenation efforts aimed at rehabilitating DO depletion sites in the Gan River can be effectively supported by targeting the removal of *in situ* microbial molecular mechanisms associated with toxic H_2_S and the potent greenhouse gas CH_4_.

## Data availability statement

The datasets presented in this study can be found in the NCBI SRA repository, accession numbers PRJNA1096924.

## Author contributions

SC: Data curation, Formal analysis, Software, Visualization, Writing – original draft, Writing – review & editing. FM: Funding acquisition, Project administration, Supervision, Writing – review & editing. YW: Project administration, Supervision, Writing – review & editing. JZ: Formal analysis, Writing – review & editing. LZ: Funding acquisition, Supervision, Writing – review & editing.

## Footnotes

^1^https://bugbase.cs.umn.edu/index.html
