# Supplementary material for: The potential linkage between sediment oxygen demand and microbes and its contribution to the dissolved oxygen depletion in the Gan River
Source: Front Microbiol. 2024 Jul 31;15:1413447. doi: 10.3389/fmicb.2024.1413447 (PMC11322766; doi:10.3389/fmicb.2024.1413447)
Supplement: Supplementary file 1 [file Data_Sheet_1.pdf]

Supplementary Material

1 Supplementary Figures and Tables

1.1 Supplementary Tables

Supplementary Table S1. Details of sampling site location and time.

Sampling site	Longitude/Latitude	Region	Distance from YZ (Km)	Sampling time
**CK**	29.92 N, 114.24 E	Up stream	5	August 3, 2022
**YZ**	29.93 N, 114.21 E	Yaozui Bridge	0	
**Down1**	29.94 N, 114.19 E	Down stream	1	
**Down2**	29.95 N, 114.19 E	Down stream	2	

Supplementary Table S2. *DO - t* standard curve of six sediment samples in Gan River.

Sample site	***DO - t***	***R^2***	***S***	***Vs /A***	***SOD ($g\cdot(m^2\cdot d)^{-1}$)***
**CK**	y = - 0.2458x + 2.9998	0.9838	0.2458	140	0.83
**YZ**	y = - 0.3632x + 3.7900	0.9688	0.3632	140	1.22
**Down1**	y = - 0.3301x + 4.3681	0.9905	0.3301	140	1.11
**Down2**	y = - 0.2721x + 2.3671	0.9913	0.2721	140	0.91

Notes: *Vs* (L) is the volume of overlying water in the cylinder; *A* (m^2) is the bottom area of a cross section of sediment in the tube cylinder.

Supplementary Table S3. Details of top5 OTUs taxon in volcano plot.

OTU	Phylum	Genus	Species
OTU1368	DTB120	*DTB120*	*unclassified DTB120*
OTU1443	Proteobacteria	*Sulfurifustis*	*unclassified Sulfurifustis*
OTU1448	Proteobacteria	*MND1*	*unclassified MND1*
OTU1473	Chloroflexi	*unclassified Anaerolineae*	*unclassified Anaerolineae*
OTU1571	Chloroflexi	*SBR1031*	*uncultured SBR1031*
OTU1712	Proteobacteria	*MND1*	*unclassified MND1*
OTU173	Chloroflexi	*SBR1031*	*unclassified SBR1031*
OTU234	Zixibacteria	*Zixibacteria*	*uncultured Zixibacteria*
OTU2883	unclassified Bacteria	*unclassified Bacteria*	*unclassified Bacteria*
OTU3075	Bacteroidota	*BSV26*	*Ignavibacteria*
OTU3408	Patescibacteria	*Gracilibacteria*	*uncultured Gracilibacteria*
OTU3492	Firmicutes	*Clostridium sensu stricto 13*	*Clostridium sensu stricto 13 Clostridium sensu stricto 13*
OTU3719	Patescibacteria	*Gracilibacteria*	*unclassified Gracilibacteria*
OTU372	Cyanobacteria	*unclassified Coleofasciculaceae*	*unclassified Coleofasciculaceae Coleofasciculaceae*
OTU3730	Proteobacteria	*Oxalobacteraceae*	*unclassified Oxalobacteraceae*
OTU4035	Desulfobacterota	*Desulfurivibrio*	*unclassified Desulfurivibrio*
OTU421	Nitrospinota	*Nitrospinaceae*	*uncultured Nitrospinaceae*
OTU5937	Firmicutes	*coprostanoligenes*	*unclassified coprostanoligenes*
OTU5988	Firmicutes	*Sedimentibacter*	*uncultured Sedimentibacter*
OTU6045	Firmicutes	*unclassified Clostridiaceae*	*unclassified Clostridiaceae*
OTU6201	Firmicutes	*Paludicola*	*Paludicola psychrotolerans*
OTU6266	Firmicutes	*Clostridium sensu stricto 12*	*uncultured Clostridium sensu stricto 12*
OTU6366	Chloroflexi	*A4b*	*unclassified A4b*

OTU	Phylum	Genus	Species
OTU6539	Firmicutes	*Caproiciproducens*	*uncultured Caproiciproducens*
OTU6546	Bacteroidota	*S15A-MN91*	*uncultured S15A-MN91*
OTU6858	Bacteroidota	*Cytophaga xylanolytica group*	*unclassified Cytophaga xylanolytica group*
OTU6995	Firmicutes	*Sedimentibacter*	*unclassified_Sedimentibacter*
OTU7146	Bacteroidota	*Bacteroides*	*Bacteriodetes bacterium enrichment culture clone*
OTU7245	Proteobacteria	*unclassified Burkholderiales*	*unclassified Burkholderiales*
OTU7256	Firmicutes	*unclassified Clostridiaceae*	*unclassified Clostridiaceae*
OTU7293	Firmicutes	*Hydrogenoanaerobacterium*	*Hydrogenoanaerobacterium saccharovorans*
OTU7384	Bacteroidota	*Bacteroides*	*unclassified Bacteroides*
OTU7403	Firmicutes	*Acidaminococcaceae*	*Acidaminococcaceae*
OTU7415	Firmicutes	*Clostridia vadinBB60 group*	*gut metagenome*
OTU7429	Firmicutes	*Clostridium sensu stricto 16*	*unclassified Clostridium sensu stricto 16*
OTU7445	Firmicutes	*unclassified Oscillospirales*	*unclassified Oscillospirales*
OTU7450	Proteobacteria	*unclassified Burkholderiales*	*unclassified Burkholderiales*
OTU7514	Bacteroidota	*FTLpost3*	*uncultured FTLpost3*
OTU791	Bacteroidota	*unclassified Microscillaceae*	*unclassified Microscillaceae*

Supplementary Table S4. Topological properties of co-occurring networks.

Network indexes	Sediment microbes with water environment factors	Sediment microbes with sediment environment factors
**Total nodes**	326	502
**Total edges**	366	621
**Average Degree**	1.974	2.474
**Network Diameter**	12	10
**Density (D)**	0.007	0.005
**Modularity**	0.568	0.5
**Average path distance**	3.617	3.507

Supplementary Table S5. The abundance and taxonomy of genus correlated with DO.

OTU	Phylum	Species	Interactions (p : positive; n : negative)	Weight
OTU7120	Firmicutes	unclassified *Clostridiaceae*	p	0.90
OTU7134	Firmicutes	unclassified *Oscillospirales*	p	0.90
OTU6995	Firmicutes	unclassified *Sedimentibacter*	p	0.89
OTU7293	Firmicutes	*Hydrogenoanaerobacterium saccharovorans*	p	0.89
OTU6951	Bacteroidota	unclassified *Alistipes*	p	0.87
OTU6386	Proteobacteria	uncultured *Ferribacterium*	p	0.87
OTU7452	Firmicutes	uncultured *Clostridium Fonticella*	p	0.86
OTU6136	Firmicutes	unclassified *Clostridia vadinBB60 group*	p	0.85
OTU6353	Firmicutes	uncultured *bacterium Acetonema*	p	0.84
OTU7190	Firmicutes	unclassified *Ruminococcaceae*	p	0.84
OTU7394	Firmicutes	uncultured *bacterium Clostridium sensu stricto 8*	p	0.83
OTU6539	Firmicutes	uncultured *Caproiciproducens*	p	0.83
OTU5760	Proteobacteria	uncultured *oc32*	n	0.93
OTU6473	Spirochaetota	uncultured *Spirochaeta*	n	0.92
OTU4024	Proteobacteria	uncultured *Sulfuritalea*	n	0.91
OTU4079	Desulfobacterota	uncultured *Desulfobulbales*	n	0.88
OTU6994	Proteobacteria	unclassified *MM2*	n	0.87
OTU4019	Acidobacteriota	unclassified *Acidobacteriae*	n	0.85
OTU6055	Acidobacteriota	uncultured *Thermoanaerobaculum*	n	0.85
OTU6917	Zixibacteria	uncultured *Nitrospinaceae*	n	0.85

OTU1735	Acidobacteriota	uncultured *Subgroup_18*	n	0.85
OTU6959	Sva0485	uncultured *Sva0485*	n	0.83
OTU5734	Sva0485	unclassified *Sva0485*	n	0.83
OTU886	Nitrospirota	uncultured *Nitrospira*	n	0.83
OTU3025	Chloroflexi	unclassified *Anaerolineae*	n	0.82

Supplementary Table S6. The abundance and taxonomy of OTUs correlated with SOD

OTU	Phylum	Species	Interactions (p : positive; n : negative)	Weight
OTU7097	Acidobacteriota	*unclassified Acidobacteriae*	p	0.79
OTU1134	Acidobacteriota	*uncultured Bryobacter*	p	0.78
OTU351	Acidobacteriota	*unclassified AKIW659*	p	0.77
OTU6445	Bacteroidota	*unclassified Microscillaceae*	p	0.87
OTU6427	Bacteroidota	*uncultured KYH767*	p	0.84
OTU6393	Bacteroidota	*uncultured env.OPS_17*	p	0.80
OTU7169	Bacteroidota	*uncultured Fluviicola*	p	0.78
OTU1012	Bacteroidota	*uncultured BSV26*	p	0.77
OTU1365	Bdellovibrionota	*uncultured Bdellovibrio*	p	0.88
OTU279	Chloroflexi	*uncultured MSBL5*	p	0.79
OTU1867	Chloroflexi	*uncultured SCGC-AB-539-J10*	p	0.77
OTU328	Chloroflexi	*uncultured GIF9*	p	0.77
OTU300	Chloroflexi	*uncultured OLB14*	p	0.76
OTU1361	DTB120	*Deltaproteobacteria GWC2_55_46*	p	0.80
OTU5754	Gemmatimonadota	*denitrifying NOB_2_F8*	p	0.81
OTU1156	Gemmatimonadota	*uncultured Gemmatimonadaceae*	p	0.78
OTU2818	Methylomirabilota	*uncultured Sh765B-TzT-35*	p	0.83
OTU1528	Myxococcota	*unclassified Blfdi19*	p	0.93
OTU7504	Myxococcota	*unclassified Myxococcaceae*	p	0.91
OTU5955	Myxococcota	*unclassified Polyangium*	p	0.84

OTU	Phylum	Species	Interactions (p : positive; n : negative)	Weight
OTU1652	Myxococcota	*uncultured mle1-27*	p	0.82
OTU5891	Myxococcota	*unclassified Anaeromyxobacter*	p	0.77
OTU1399	Myxococcota	*uncultured Anaeromyxobacter*	p	0.76
OTU5895	Nitrospirota	*uncultured 4-29-1*	p	0.77
OTU8099	Nitrospirota	*uncultured Nitrospira*	p	0.77
OTU6410	Proteobacteria	*unclassified Rhodocyclaceae*	p	0.98
OTU7031	Proteobacteria	*uncultured Azohydromonas*	p	0.91
OTU6594	Proteobacteria	*uncultured JG36-GS-52*	p	0.87
OTU5959	Proteobacteria	*uncultured KF-JG30-C25*	p	0.82
OTU5869	Proteobacteria	*unclassified Acetobacteraceae*	p	0.81
OTU6312	Proteobacteria	*unclassified BD1-7_clade*	p	0.80
OTU6877	Proteobacteria	*unclassified Ellin6067*	p	0.80
OTU6700	Proteobacteria	*unclassified Denitratisoma*	p	0.79
OTU6832	Proteobacteria	*uncultured CCM19a*	p	0.79
OTU1003	Proteobacteria	*uncultured Curvibacter*	p	0.79
OTU1683	Proteobacteria	*uncultured Nitrosomonas*	p	0.77
OTU6578	Proteobacteria	*unclassified Gallionellaceae*	p	0.77
OTU1648	Proteobacteria	*uncultured GOUTA6*	p	0.77
OTU1312	Proteobacteria	*unclassified IS-44*	p	0.77
OTU1697	Proteobacteria	*uncultured Rhodoferax*	p	0.76
OTU670	Proteobacteria	*uncultured pLW-20*	p	0.76

**OTU**	**Phylum**	**Species**	**Interactions (p : positive; n : negative)**	**Weight**
OTU1702	Proteobacteria	*unclassified MND1*	p	0.76
OTU5874	Sva0485	*uncultured Spirochaetales*	p	0.79
OTU227	unclassified Bacteria	*unclassified Bacteria*	p	0.92
OTU5567	Bacteroidota	*uncultured BD2-2*	n	0.76
OTU7315	Chloroflexi	*unclassified Anaerolineaceae*	n	0.80
OTU7456	Chloroflexi	*uncultured RBG-16-58-14*	n	0.76
OTU5812	Cyanobacteria	*Ceratophyllum demersum*	n	0.83
OTU7415	Firmicutes	*gut metagenome*	n	0.83
OTU7328	Firmicutes	*unclassified Lachnospiraceae*	n	0.79
OTU7239	Firmicutes	*unclassified Clostridium sensu stricto 1*	n	0.76
OTU3730	Proteobacteria	*unclassified Oxalobacteraceae*	n	0.82
OTU6126	Proteobacteria	*unclassified Rhodocyclaceae*	n	0.79
OTU1527	Proteobacteria	*uncultured Arenimonas*	n	0.78
OTU7266	Proteobacteria	*unclassified Hydrogenophilaceae*	n	0.76
OTU5799	Spirochaetota	*metagenome Treponema*	n	0.81

1.2 Supplementary Figures

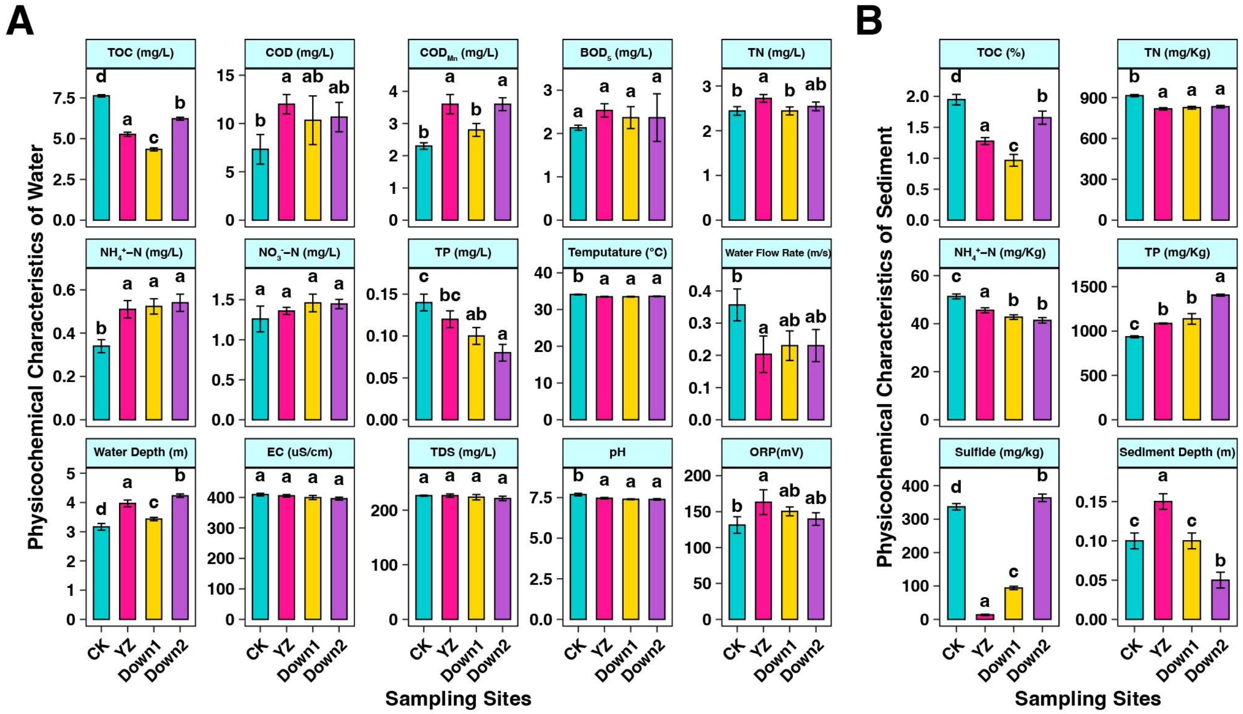

Supplementary Figure S1. Summary of environmental variables of sampled river. **(A)** Concentration of TOC, COD, COD_{Mn}, BOD_5, TN, NH_4^+-N, NO_3^--N, TP, Temperature, water flow rate, water depth, EC, TDS pH and ORP in water. **(B)** Concentration of TOC, TN, NH_4^+-N, TP, sulfide and sediment depth in sediments.

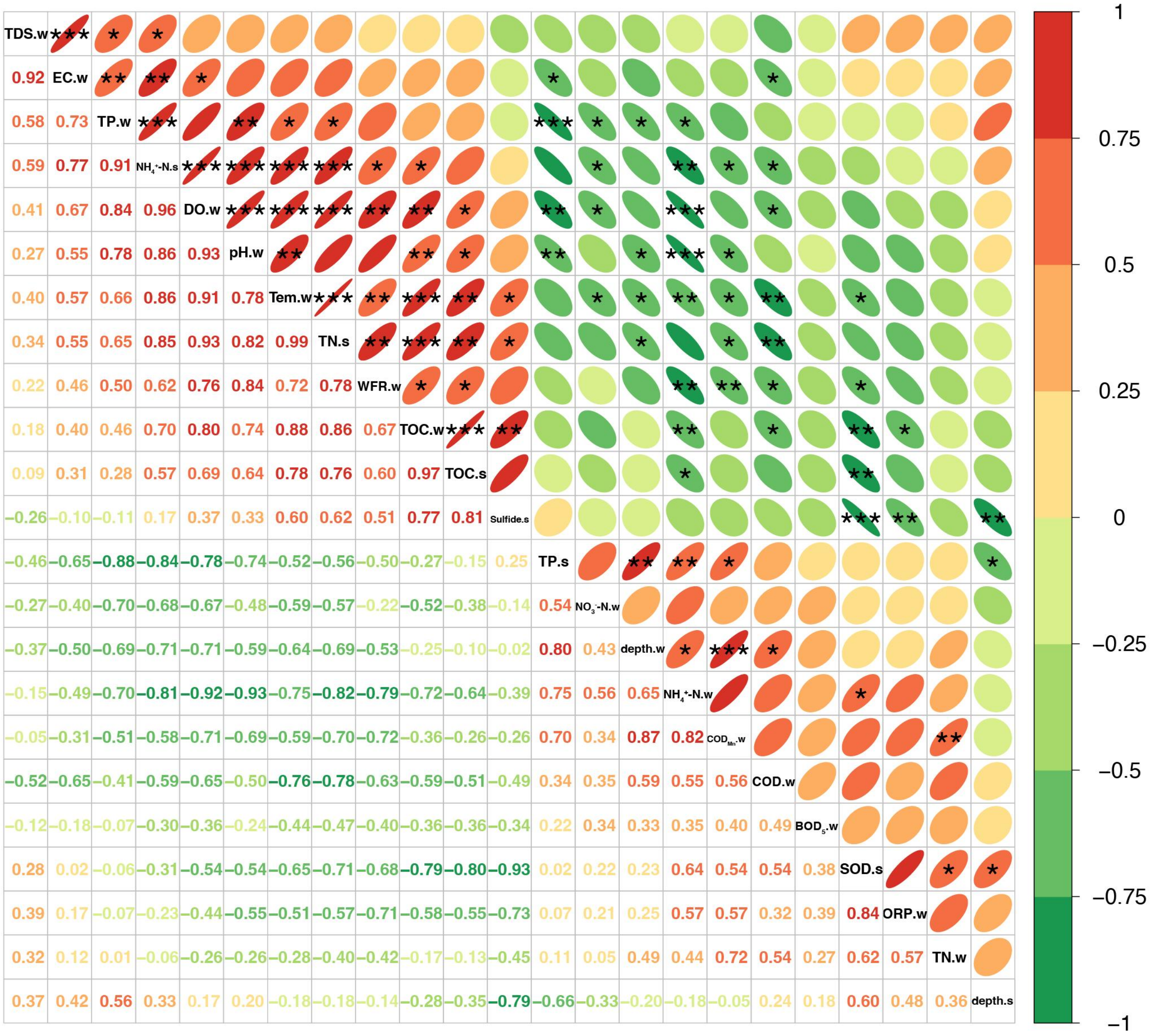

Supplementary Figure S2. Associations between physicochemical parameters of water (.w) and sediments (.s). Circle colors of upper triangular indicates the r-value of the Spearman correlations : red, positive; green, negative. "*" represents the degree of significance : *, $p<0.05$; **, $p<0.01$; ***, $p<0.001$. Numbers of lower triangular represents the degree of relationship : 1, positive relationship; -1, negative relationship.

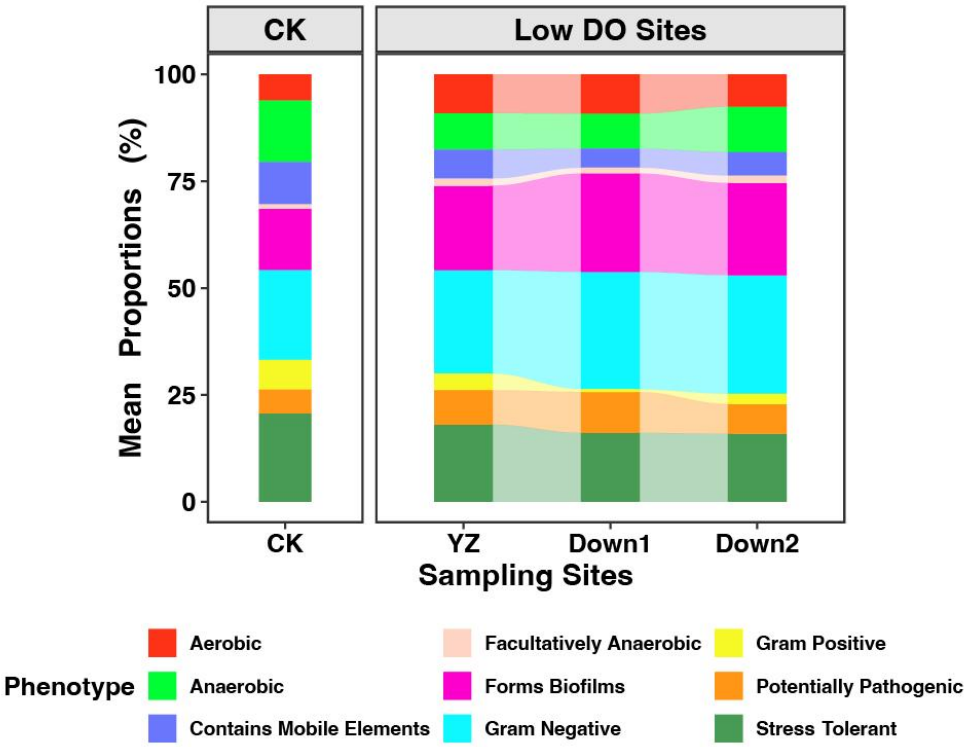

Supplementary Figure S3. The mean proportions (%) of Phenotype predicted by BugBase.

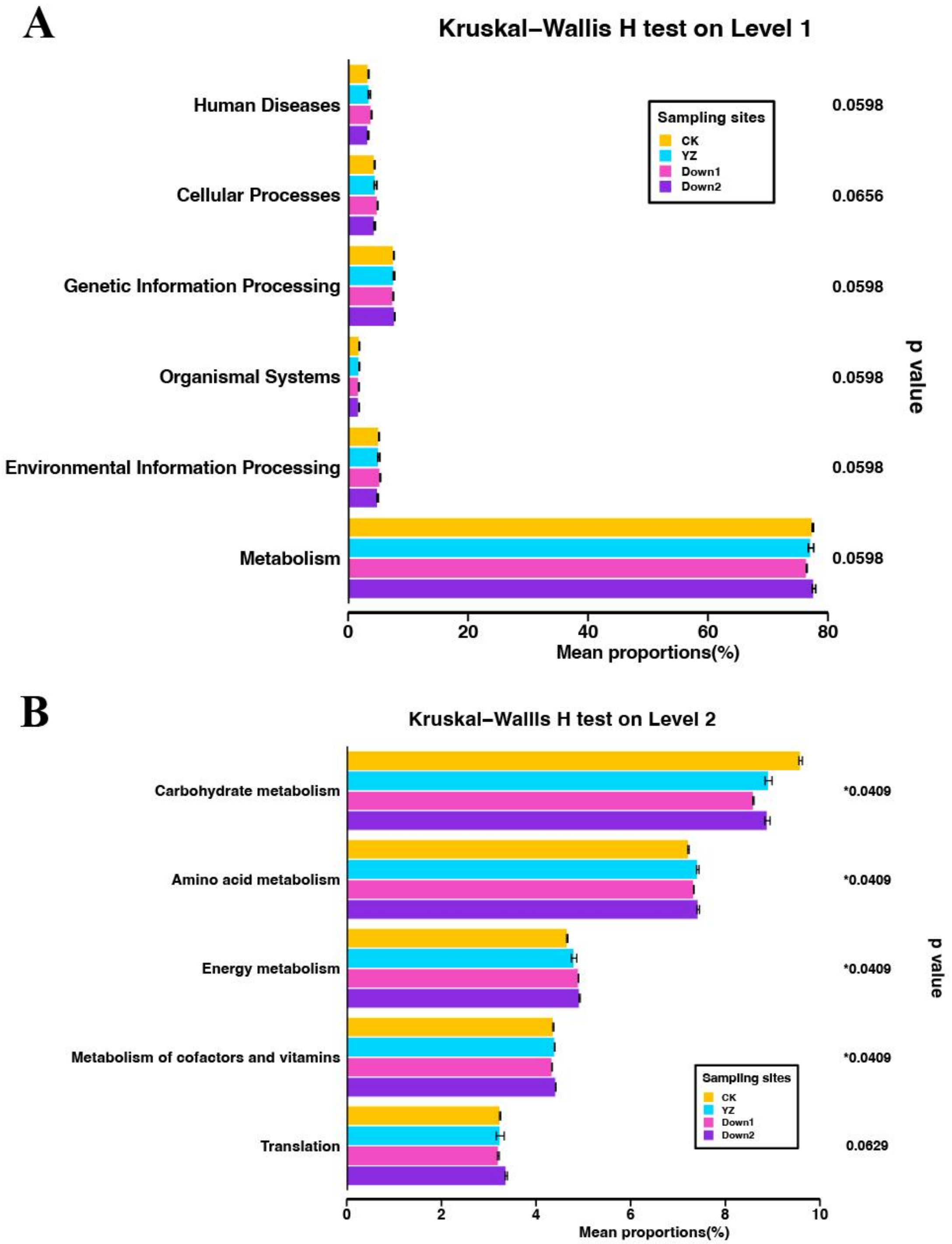

Supplementary Figure S4. The relative abundance of differential functional categories (KEGG) of bacterial communities using PICRUSt2 analysis at pathway level 1 **(A)**, and pathway level 2 **(B)** in sediment samples (*p <0.05, **p <0.01 ,***p <0.001).
